# Isoform-Selective PAD2/PAD4 Substrates with Unnatural Amino Acids Enable Cellular Peptidylarginine Deiminase Activity Profiling and Reveal Vimentin Citrullination Effects in Macrophages

**DOI:** 10.1021/acs.biochem.5c00391

**Published:** 2025-09-25

**Authors:** Oliwia Gorzeń, Agata Mikołajczyk-Martinez, Abdulla Al. Mamun, Natalia Horbach, Olha Severynovska, Grzegorz Bereta, Ewa Bielecka, Piotr Mydel, Marcin Drąg, Tomasz Kantyka, Marcin Poręba

**Affiliations:** 1 Faculty of Chemistry, Wroclaw University of Science and Technology, Wroclaw 50-370, Poland; 2 Faculty of Veterinary Medicine, Wroclaw University of Environmental and Life Sciences, Wroclaw 50-375, Poland; 3 Malopolska Centre of Biotechnology, 37799 Jagiellonian University, Krakow 30-387, Poland; 4 Faculty of Biochemistry, Biophysics and Biotechnology, Jagiellonian University, Krakow 30-387, Poland; 5 Broegelmann Research Laboratory, University of Bergen, Bergen NO-5020, Norway; 6 Centre for Chemical Biology, Institute of Physical Chemistry, Polish Academy of Sciences, Warsaw 01-224, Poland; 7 Faculty of Medicine, Wroclaw University of Science and Technology, Wroclaw 51-377, Poland

## Abstract

Peptidylarginine deiminases (PADs) catalyze the calcium-dependent conversion of arginine to citrulline, which affects diverse cellular processes. Among the human PAD isoforms, PAD2 and PAD4 are particularly relevant because of their distinct tissue distributions and substrate preferences. However, the lack of isoform-selective substrates has limited our ability to discriminate between their activities in biological systems. In this study, we developed PAD2- and PAD4-selective fluorogenic peptide substrates using the Hybrid Combinatorial Substrate Library (HyCoSuL) strategy, which incorporates both natural and over 100 unnatural amino acids. Substrate specificity profiling at P4–P2 positions revealed that PAD2 tolerates a broader range of residues, particularly at the P2 position, whereas PAD4 displays more selective preferences, favoring aspartic acid at this site. Based on these insights, we designed and validated peptide substrates with high selectivity for PAD2 or PAD4, enabling isoform-specific kinetic analysis *in vitro*. We demonstrated the utility of these substrates in profiling PAD activity in THP-1 macrophages, revealing dominant PAD2 activity in PMA (phorbol 12-myristate 13-acetate)/LPS (lipopolysaccharide)-stimulated monocytes. Furthermore, PAD4-mediated citrullination of vimentin modulates its susceptibility to caspase and calpain cleavage, potentially altering its function as a damage-associated molecular pattern (DAMP). Our findings provide a framework for the development of PAD-selective inhibitors and chemical probes, enabling the precise dissection of isozyme-specific PAD functions in health and disease.

## Graphical abstract

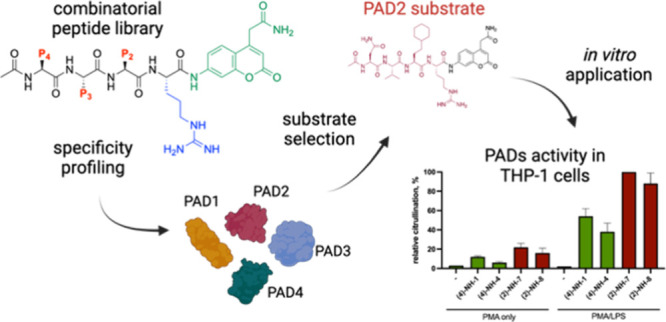

## Introduction

Peptidylarginine deiminases (PADs) are a family of calcium-dependent enzymes that post-translationally convert positively charged arginine residues into neutral citrulline, a modification known as citrullination.^1,2^ This modification irreversibly alters the target protein by removing the positive charge of arginine, thereby changing its hydrogen-bonding capacity and overall structure. Consequently, PADs activity can significantly impact protein–protein interactions, signaling pathways, and immunogenicity, positioning these enzymes as key regulators in multiple physiological and pathological contexts.^3–6^ Citrullination has been described in several diseases, particularly autoimmune, inflammatory, and neurodegenerative conditions, as well as in cancer progression. It plays a pivotal role in the etiology of rheumatoid arthritis (RA), where abnormal protein citrullination leads to an immunological reaction and the appearance of anticitrullinated antibodies, which trigger a cascade of inflammation.^7,8^ There are five PAD isoforms in humans (PAD1–4 and PAD6), each with distinct tissue distributions and functional roles in cellular differentiation, nerve growth, apoptosis, inflammation, gene regulation, and early development.^1,9^ Among the PAD family members, PAD2 and PAD4 have garnered particular attention because of their abundance and distinct localizations.^10,11^ PAD2 is broadly expressed (e.g., in muscle, brain, and macrophages) and acts on a wide array of substrates, including cytoskeletal proteins (such as vimentin and actin), fibrinogen and other proteins in blood, various cytokines, and even histones.^12^ In contrast, PAD4 is primarily nuclear (notably in granulocytes and other immune cells) and is best known for citrullinating histones, a process that influences chromatin structure and gene expression.^12,13^ Through histone citrullination, PAD4 plays a crucial role in chromatin decondensation, for instance, during the formation of neutrophil extracellular traps (NETs) as part of the innate immune response.^14^ Biochemical studies indicate that PAD4 has a more restrictive substrate specificity than PAD2, which exhibits a broader tolerance for different arginine contexts. For example, when both enzymes were tested on the same protein (fibrinogen) *in vitro*, PAD2 citrullinated more arginine sites than PAD4.^15^ This difference in selectivity suggests that PAD2 is a more promiscuous citrullinating enzyme, whereas PAD4 targets specific proteins in specialized pathways. Identifying suitable substrates is essential for characterizing PAD activity and developing selective assays for PADs. Historically, simple arginine-containing compounds have been used as surrogate substrates for PAD.^16^ For example, *N*α-benzoyl-l-arginine ethyl ester (BAEE) is a small molecule that is converted into citrulline and ammonia upon citrullination by PAD, which can be detected using a colorimetric reagent.^17,18^ These assays provide early quantification of PAD activity, although they do not replicate the full sequence context of natural protein substrates. To better emulate physiological targets, researchers have also employed protein-derived peptides (e.g., fragments of histone tails or fibrin) as PAD substrates, which allows assessment of how neighboring amino acids influence enzyme efficiency and isoform preference.^15^ Moreover, high-throughput approaches using peptide libraries have been applied to systematically probe PAD substrate specificity, confirming distinct sequence preferences for PAD2 and PAD4.^16^ In parallel with substrate development, substantial effort has been focused on PAD inhibitors as both research tools and potential therapeutics.^19–22^ Many potent inhibitors feature electrophilic warheads that irreversibly alkylate the active site cysteine, thereby inactivating the enzyme. The prototypical compound of this class is Cl-amidine, a haloacetamidine-containing arginine analog that covalently modifies PADs and suppresses PAD activity in cells and *in vivo.* ^17^ Although these pan-PAD inhibitors have shown efficacy in preclinical models of RA and ulcerative colitis, isoform selectivity remains a challenge. Recent advances, including structure-guided design and high-throughput screening, have yielded selective inhibitors such as GSK484 (PAD4)^23^ and AFM-30a (PAD2).^24^ The availability of both irreversible and reversible PAD inhibitors with isoform selectivity provides versatile tools for modulating citrullination in biological systems. Another important set of chemical tools for PAD research are activity-based probes (ABPs), which are modified PAD inhibitors equipped with a detectable tag.^25–27^ ABPs are designed to covalently bind to active PAD enzymes, labeling them for visualization or enrichment. For example, fluorescently labeled PAD inhibitors (e.g., FITC-conjugated F-amidine analogs) have been used to image active PAD4 in cells, while biotin-conjugated probes enable the pull-down of PAD4 and its associated proteins from cell lysates.^4^ These probes are invaluable for profiling enzyme activity *in situ* and can be used in competitive assays to evaluate the selectivity of new PAD inhibitors based on their ability to block probe labeling. Recent advances have introduced “clickable” ABPs with bioorthogonal handles and improved cell permeability, such as second-generation probes based on optimized warheads like BB-Cl-amidine, to further enhance the sensitivity and versatility of PAD activity profiling.^27^ While significant progress has been made in elucidating PAD biology and developing chemical tools, current methods do not always permit the clear discrimination of individual PAD isozyme activities. In particular, there is a scarcity of substrates that are highly selective for PAD2 and PAD4. In this study, we employed the Hybrid Combinatorial Substrate Library (HyCoSuL) approach, a peptide-based platform incorporating both natural and unnatural amino acids,^28,29^ to systematically profile the substrate specificity of PAD2 and PAD4. By leveraging the expanded chemical diversity offered by unnatural residues, we aim to overcome the limitations of conventional peptide libraries and identify isoform-selective substrates that can distinguish between PAD2 and PAD4 activities. These tailored substrates will enable the precise detection of individual PAD isoforms in complex biological systems, providing critical tools to dissect their distinct roles in health and disease and accelerate diagnostic and therapeutic development.

## Materials and Methods

### Reagents, Antibodies, and Enzymes

All chemicals were sourced from commercial suppliers and used without further purification. Fmoc- and Boc-protected amino acids were purchased from various vendors, including Combi-Blocks, Iris Biotech GmbH, Angene, Ambeed, and Merck Sigma-Aldrich. The fluorescent dye Fmoc-ACC–OH was synthesized according to the procedure described by Maly et al.^30^ Rink amide AM resin (200–300 mesh, loading 0.74 mmol/g) for substrate synthesis was obtained from Iris Biotech GmbH. Coupling reagents, such as HATU and HBTU, along with piperidine, trifluoroethanol (TFE), and trifluoroacetic acid (TFA), were purchased from Iris Biotech GmbH. Anhydrous HOBt was obtained from Creosalus, and other reagents, including 2,4,6-collidine, acetonitrile (ACN), and triisopropylsilane (TIPS) were sourced from Merck Sigma-Aldrich. The solvents and reagents used, including N,N-dimethylformamide (DMF, pure for analysis), methanol (MeOH), dichloromethane (DCM), acetic acid (AcOH), diethyl ether (Et_2_O), and phosphorus pentoxide (P_2_O_5_), were obtained from POCh (Gliwice, Poland). All substrates were purified via reverse-phase HPLC using a Waters system (comprising a Waters M600 solvent delivery module and Waters M2489 detector system) with a semipreparative Discovery C8 column (particle size 10 μm). The purity and molecular mass of the compounds were confirmed using a Waters LC-MS system. The antibodies used in this study were as follows: antivimentin (Cell Signaling, 5741), anticitrullinated vimentin (Cayman, 22054), anti-GSDMD (Cell Signaling, 97558), anti-NLRP3 (Cell Signaling, 15101), anticaspase-1 (full length) (Cell Signaling, 3866), and anticaspase-1 (p10 subunit) (Cell Signaling, 89332). Caspase-3 was a kind gift from Prof. Guy Salvesen (SBP Medical Discovery Institute, La Jolla, USA). Calpain-1 (208713) and trypsin (T6424) were purchased from Merck Sigma-Aldrich. Vimentin was purchased from Biotechne (NBP2- 35139). PAD4 was expressed and purified as described previously.^31^ PAD1 (10784), PAD2 (10785), and PAD3 (10786) were purchased from Cayman Chemicals.

### Enzyme Kinetic Studies

All kinetic studies were conducted using an fMax fluorescence plate reader (Molecular Devices) operating in the fluorescence kinetic mode with 96- or 384-well plates. The fluorescence of ACC was monitored at excitation and emission wavelengths of 355 and 460 nm, respectively. The assay buffers for PADs and trypsin consisted of 100 mM TRIS-HCl, 10 mM NaCl, 5 mM CaCl_2_, and 5 mM DTT (pH 7.2). All experiments, including library screening and substrate kinetics, were repeated at least three times and the average values were reported. Kinetic data were analyzed using GraphPad Prism software (version 10.2.0).

### PAD Substrate Specificity Profiling

The P1-Arg HyCoSuL library was employed to profile the substrate specificity of PADs and trypsin at the P4, P3, and P2 positions, according to the Berger and Schechter nomenclature.^32^ Each sublibrary (P4, P3, and P2) was individually screened at a final substrate concentration of 100 μM in a 100 μL reaction volume. PAD2 was used at concentrations of 150 nM, and PAD4 at 75 nM. To evaluate citrullination kinetics, the substrates were incubated with trypsin alone or with PAD. Incubation with PAD results in the citrullination of arginine (Arg) to citrulline (Cit), which consequently reduces the cleavage efficiency of Arg-containing substrates by trypsin. Screening was performed for a total duration of 30 min. The difference in substrate hydrolysis between the trypsin-only and trypsin+PAD conditions was calculated over time to determine the rate of citrullination. Each library was screened in triplicate, and the average values were used to construct a PAD substrate specificity matrix. The standard deviation for each substrate was less than 15%. Notably, substrates that were not cleaved by trypsin could not be evaluated for PAD specificity, as no measurable signal was generated. The citrullination rate for the most efficiently recognized amino acid at each position was normalized to 100%, with corresponding adjustments made for the other residues. Additionally, PAD1 and PAD3 substrate specificities were evaluated using a pool of natural amino acids under the same conditions. PAD1 was used at concentrations of 125 nM, and PAD3 at 200 nM.

### Fluorescent Substrate Synthesis

ACC-containing peptide substrates were synthesized using standard solid-phase peptide synthesis (SPPS) on Rink-amide resins. The resin was initially swollen in dichloromethane (DCM), washed with dimethylformamide (DMF), and the Fmoc protecting group was removed using 20% piperidine in DMF. Fmoc-ACC–OH was preactivated with 1-hydroxybenzotriazole (HOBt) and *N,N’*-diisopropylcarbodiimide (DICI) in DMF and subsequently coupled to the resin. The coupling step was repeated to ensure the process was complete. Following deprotection of the ACC moiety, Fmoc-protected amino acids were sequentially coupled to elongate the peptide chain using HATU/2,4,6-collidine in DMF, with each coupling step being followed by Fmoc deprotection. The N-terminus of the peptide was acetylated using a mixture of acetic acid (AcOH), HBTU, and *N,N*-diisopropylethylamine (DIPEA) in DMF. The peptides were cleaved from the resin using a trifluoroacetic acid (TFA)/triisopropylsilane (TIPS)/water cleavage cocktail (95:2.5:2.5, v/v/v), precipitated with cold diethyl ether, and lyophilized. Crude products were purified using reverse-phase high-performance liquid chromatography (RP-HPLC), and their molecular weights were confirmed using liquid chromatography–mass spectrometry (LC-MS). The final purified substrates were dissolved in dimethyl sulfoxide (DMSO) at a concentration of 20 mM for subsequent use.

### Kinetic Analysis of Fluorescent Substrates

Substrate screening for PAD2 and PAD4 was performed at a final substrate concentration of 10 μM, as described in “PAD substrate specificity profiling section”. PAD2 and PAD4 were tested across a concentration range of 37.5 nM to 300 nM, whereas the trypsin concentration was 5 nM. Briefly, each substrate was preincubated with the specified concentration of PAD in a 50 μL reaction volume (or in 50 μL of buffer for PAD-untreated control) for 15 min, after which 50 μL of trypsin was added to the reaction wells. The hydrolysis of Arg-containing substrates by trypsin was monitored over time. The percentage of substrate citrullination was calculated by comparing the extent of trypsin-mediated cleavage of untreated substrates with that of substrates preincubated with PAD. Each assay was performed in triplicate, and the resulting citrullination percentages are presented graphically. The standard deviation of each data point was less than 15%.

### Protein Analysis of PMA-Differentiated THP-1 Cells

Low-passage THP-1 cells were seeded into 12-well plates and treated with increasing concentrations of phorbol 12-myristate 13-acetate (PMA) ranging from 1 nM to 100 nM. The cells were incubated for 24 h to allow adherence and differentiation. Following incubation, the cells were lysed, and the lysates were subjected to SDS-PAGE (200 V, 30 min) and Western blot analysis. The expression and cleavage of vimentin, gasdermin D (GSDMD), NLRP3, and caspase-1 were analyzed using specific primary antibodies (1:500–1:1,000), followed by incubation with an antirabbit secondary antibody conjugated to Alexa Fluor 800 (1:10,000). Fluorescence signals were detected using an Azure Biosystems Sapphire RGB-NIR biomolecular imager. Ponceau S staining was used as a loading control to ensure equal protein loading in each lane. In a subsequent experiment, THP-1 cells were pretreated with specific inhibitors for 1 h prior to PMA-induced differentiation (40 nM PMA). The inhibitors used were: BAPTA (10 μM, calcium chelator), calpeptin (100 μM, calpain inhibitor), 4-bisindolylmaleimide (25 μM, PKC inhibitor), N-α-benzoyl-N5-(2-chloro-1-iminoethyl)-l-Orn amide or Cl-amidine (25 μM, pan PAD inhibitor, PADi), Z-DEVD-FMK (10 μM, caspase-3 inhibitor), and VX-765 (10 μM, caspase-1 inhibitor). After PMA treatment, the cells were lysed, and the lysates were processed for SDS-PAGE (200 V, 30 min) and Western blotting. Protein detection and visualization were performed in the same manner as in the cell-based experiments without inhibitors.

### Vimentin Citrullination Assay and Processing

Recombinant vimentin (80 μg) was incubated with 100 nM PAD4 for varying durations (5–60 min). Following incubation, the samples were subjected to SDS-PAGE, and the proteins were subsequently transferred onto a nitrocellulose membrane. Western blot analysis was performed using antivimentin and anticitrullinated vimentin antibodies, followed by a secondary antibody conjugated to Alexa Fluor 800 to assess the time-dependent decrease in native vimentin levels and the corresponding increase in citrullinated vimentin levels. Subsequently, both native and citrullinated vimentin were incubated with either human recombinant caspase-3 (20 nM) or calpain-1 (10 nM) for 30 min. After incubation, the samples were analyzed by SDS-PAGE followed by colloidal Coomassie dye G-250 (Thermo, 24590) staining.

### Analysis of the Influence of Citrullination and Proteolytic Processing on Vimentin Activity as a Damage-Associated Molecular Pattern

THP-1 cells were seeded and stimulated with PMA and LPS, as described in “Protein analysis of PMA-differentiated THP-1 cells” section. After stimulation, vimentin, citrullinated vimentin, or their cleavage products-generated by caspase-3 or calpain-1 digestion were added to the cells, which were then incubated for an additional 48 h. Following incubation, lactate dehydrogenase (LDH) activity in the supernatant was measured using the CytoTox 96 Non-Radioactive Cytotoxicity Assay (G1718) as an indicator of membrane disruption, according to the manufacturer’s protocol. Cytotoxicity was calculated as the percentage of LDH released from the treated samples relative to the LDH release induced by the lysis buffer and normalized to the spontaneous release from the control cells. Absorbance was measured at 490 nm using a spectrophotometric microplate reader (Gemini XPS, Molecular Devices). All experiments were independently performed in triplicate. Statistical analyses were performed using GraphPad Prism software (GraphPad Software, Inc., La Jolla, CA, USA). One-way ANOVA followed by Tukey’s multiple comparisons posthoc test was used to determine statistical significance. For Western blot analysis, proteins from the cell lysates were prepared as described in “Protein analysis of PMA-differentiated THP-1 cells”. To isolate proteins from the supernatant, 1 mL of the sample was mixed with 0.5 mL of 30% trichloroacetic acid (TCA) at room temperature and incubated on ice for 30 min. The mixture was centrifuged at 12,000 × g for 20 min at 4 °C. The supernatant was discarded, and the pellet was washed with 100 μL of 10% TCA, followed by centrifugation. The pellet was then washed with 300 μL of acetone, centrifuged again, and allowed to dry completely. The remaining residues were dissolved in SDS-PAGE loading buffer under reducing conditions. Proteins of interest were detected using primary antibodies against GSDMD and interleukin-1β (IL-1β), followed by secondary antirabbit antibodies conjugated with Alexa Fluor 800. Fluorescence signals were visualized using an Azure Biosystems Sapphire RGB-NIR biomolecular imager. Ponceau S staining was used as a loading control to verify equal protein loading in the SDS-PAGE.

### Analysis of PAD Activity in THP-1 Cells

Low-passage THP-1 cells were seeded into 12-well plates and treated with PMA (40 nM) to induce differentiation. Following differentiation, the cells were treated with lipopolysaccharide (LPS, 1 μg/mL) overnight to activate the inflammatory signaling. The next day, cells were harvested, and lysates were prepared in PAD assay buffer (excluding DTT) by sonication, followed by centrifugation (10 min, 12,000 × g, 4 °C) to remove cellular debris. The resulting supernatants were adjusted to a total protein concentration of 5 mg/mL, supplemented with DTT (10 mM), and incubated with PAD peptide substrates (20 μM) for 30 min. Trypsin was then added to selectively hydrolyze the noncitrullinated substrates. In the control wells, the same substrates were incubated in buffer alone for 30 min before adding trypsin at the same concentration. Citrullination levels were calculated by comparing trypsin-mediated hydrolysis in PAD-treated and control samples. The most efficiently citrullinated substrate was set to 100%, and the citrullination levels of the other substrates were adjusted proportionally.

## Results

### Kinetic Assay for PAD Specificity Profiling

A range of methods has been developed to assess the activity and substrate specificity of peptidylarginine deiminases (PADs).^33–35^ Classical assays include colorimetric detection of citrulline (e.g., via diacetyl monoxime), high-performance liquid chromatography (HPLC)-based assays, antibody-based platforms such as ABAP (antibody-based anticitrullinated peptide ELISA), and more recently, real-time spectrophotometric and fluorescence-based assays. Among these, fluorescence-based assays have gained prominence because of their sensitivity, scalability, and compatibility with real-time kinetics. For instance, Wildeman and Pires developed a PAD4 assay using a fluorogenic substrate (Z-Arg-AMC), where PAD-mediated citrullination of arginine rendered the substrate resistant to trypsin cleavage, thus blocking fluorophore release and fluorescence increase.^33^ This principle, the loss of trypsin recognition following citrullination, offers a highly convenient strategy for monitoring PAD activity. Building on this principle, we developed a kinetic assay using a diverse panel of tetrapeptide substrates with the general structure Ac-P4-P3-P2-Arg-ACC (HyCoSuL),^36,37^ aligned with the Schechter and Berger nomenclature.^32^ Instead of the AMC fluorophore used in previous studies,^33^ we employed 7-amino-4-carbamoylmethylcoumarin (ACC), which exhibits similar properties in terms of fluorescence quenching and release upon proteolytic cleavage but is better suited for the synthesis of peptide libraries and higher signal stability.^29^ In this assay, each substrate was incubated under two parallel conditions: one with trypsin alone (control) and the other with a combination of PAD and trypsin. PAD-mediated conversion of arginine (Arg) to citrulline (Cit) prevents trypsin from cleaving the amide bond between P1 and the fluorophore, thus diminishing fluorescence (Figure 1). The kinetic difference between the two conditions reflected the rate of citrullination. This setup allows real-time measurement of citrullination kinetics, providing both temporal and positional specificity across P4–P2 via HyCoSuL. Notably, when PAD activity is slow and cannot compete with immediate trypsin cleavage, a sequential protocol is used, in which substrates are preincubated with PAD, followed by delayed trypsin addition. This ensures that slower-reacting PAD isoforms (PAD1 and PAD3) or lower activity conditions are still captured with high fidelity. Compared to earlier fluorescence-based PAD assays, our approach offers broader specificity profiling and higher resolution owing to the combinatorial peptide library design. This approach bridges the biochemical sensitivity of fluorogenic assays with the specificity mapping potential of positional substrate screening, thus serving as a versatile platform for both fundamental enzyme profiling and high-throughput screening of PAD inhibitors.

**Figure 1:**
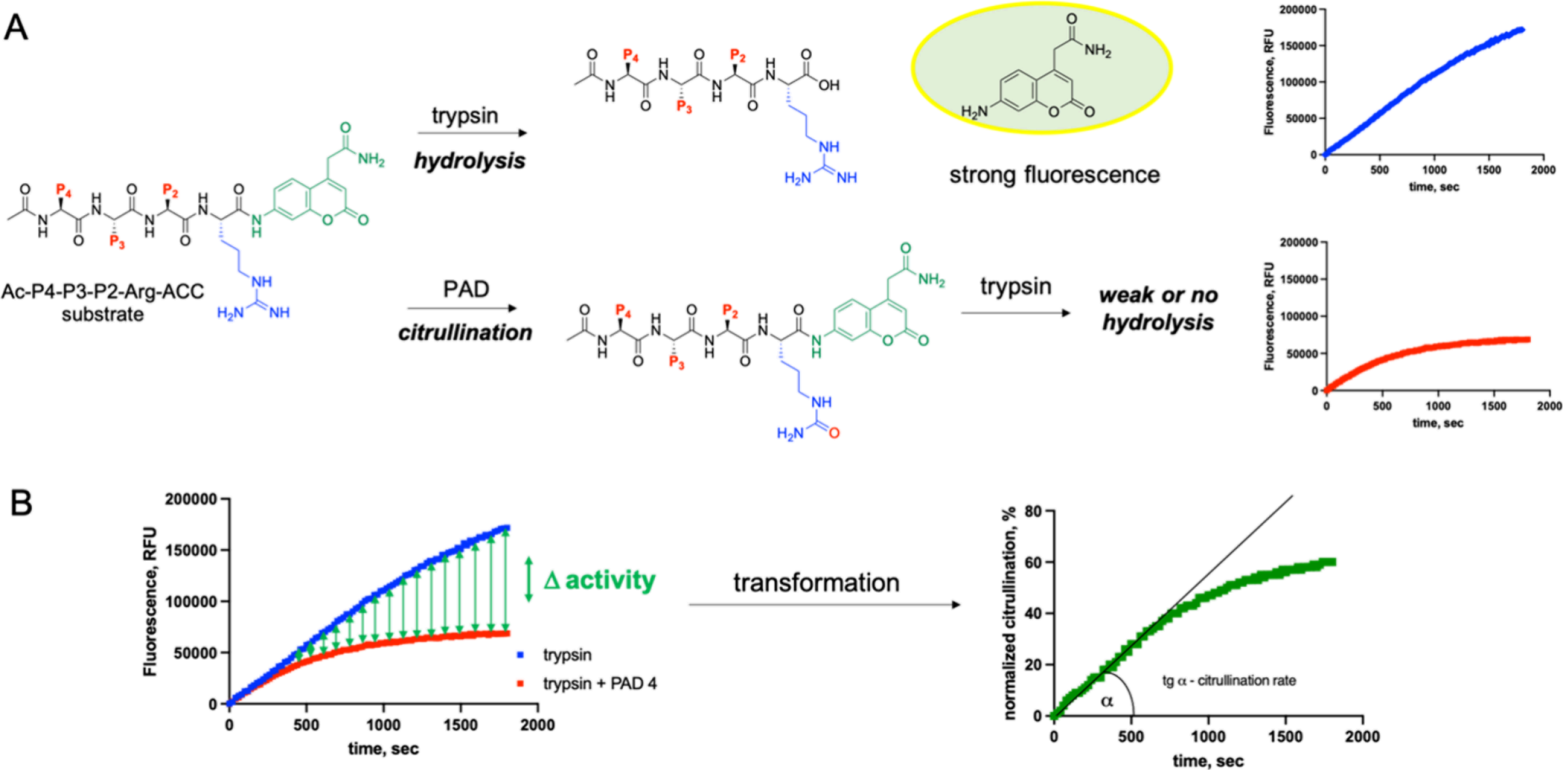
Overview of the analysis of PAD substrate specificity using fluorogenic peptide substrates.(A) A peptide library with the general structure Ac-P4-P3-P2-Arg-ACC was used to assess citrullination activity. In the control assay, peptides were directly incubated with trypsin, which cleaves at the P1-Arg residue, releasing the ACC fluorophore and resulting in a progressive increase in fluorescence over time. In the experimental assay, peptides are first treated with PAD to induce citrullination at the P1-Arg site, thereby reducing susceptibility to subsequent trypsin cleavage and diminishing the fluorescence output. (B) Fluorescence kinetics of the trypsin-only and PAD/trypsin-treated reactions were compared. The citrullination rate was inferred from the reduction in fluorescence intensity, reflecting the extent of Arg-to-citrulline conversion at the cleavage site.

### Trypsin Substrate Specificity At the P4–P2 Positions

Trypsin is a well-established serine protease widely used in proteomics because of its reliable cleavage specificity and broad substrate tolerance.^38^ It hydrolyzes peptide bonds at the carboxyl side of arginine and lysine residues and is highly compatible with sequence-diverse proteolytic workflows.^39^ Owing to its broad and well-characterized specificity, trypsin was selected as the reporter enzyme in our fluorescent kinetic assay. In our experimental design, fluorogenic tetrapeptide substrates were cleaved by trypsin only if the P1 arginine remained unmodified. Citrullination of this arginine by PAD enzymes converts it to citrulline, which is no longer recognized by trypsin, thereby preventing cleavage and subsequent fluorescence release. This loss of signal serves as a direct readout for citrullination. However, this method relies on the prerequisite that the peptide substrate is a target for trypsin. If the substrate is poorly or not at all recognized by trypsin, a lack of fluorescence signal cannot be attributed to citrullination, making it essential to first assess the positional specificity of trypsin. To determine the compatibility of different peptide sequences with trypsin, we performed a systematic analysis using the P1-Arg HyCoSuL library (Figure 2). This allowed us to map the substrate preferences of trypsin at the P2, P3, and P4 positions. The results showed that at the P2 position, trypsin recognizes almost all natural amino acids. The highest preferences were observed for alanine, serine, and threonine, whereas the lowest activity was detected for arginine and lysine. Nonetheless, even these basic amino acids were cleaved to a measurable extent, making them suitable for inclusion in PAD specificity studies. In contrast, trypsin does not recognize several amino acid types at the P2 position, including D-amino acids, dehydroamino acids, Abz-derivatives, and several bulky unnatural residues such as Bip, Bpa, Phe­(F_5_), and Phe­(guan). These substrates cannot be used to analyze PAD specificity because they do not yield any signal in the absence of PAD activity. At the P3 position, trypsin exhibited a broader specificity. It recognizes a wide variety of amino acids, including d-enantiomers and bulky residues. However, a few notable exceptions were noted. Proline, thiazolidine (Thz), piperidine (Pip), and *tert*-leucine (Tle) were not cleaved by trypsin at P3 and were excluded from further analysis. All other amino acids, even those with relatively low activity (down to 10% of the best recognized residue (His (Bzl))), were acceptable. The P4 position exhibited the broadest recognition profile. Trypsin recognizes virtually all tested amino acids at this position, regardless of their polarity, size, or stereochemistry. This finding makes the P4 position highly flexible for substrate-design and screening. In conclusion, P1-Arg HyCoSuL analysis revealed that trypsin possesses a broad and useful specificity profile at the P4–P2 positions, making it a suitable enzyme for use in our fluorescent PAD assay. With a few exceptions, where certain residues are not cleaved, trypsin enables accurate analysis of citrullination kinetics and substrate preferences, supporting its use as the core component of this specificity-profiling platform.

**Figure 2:**
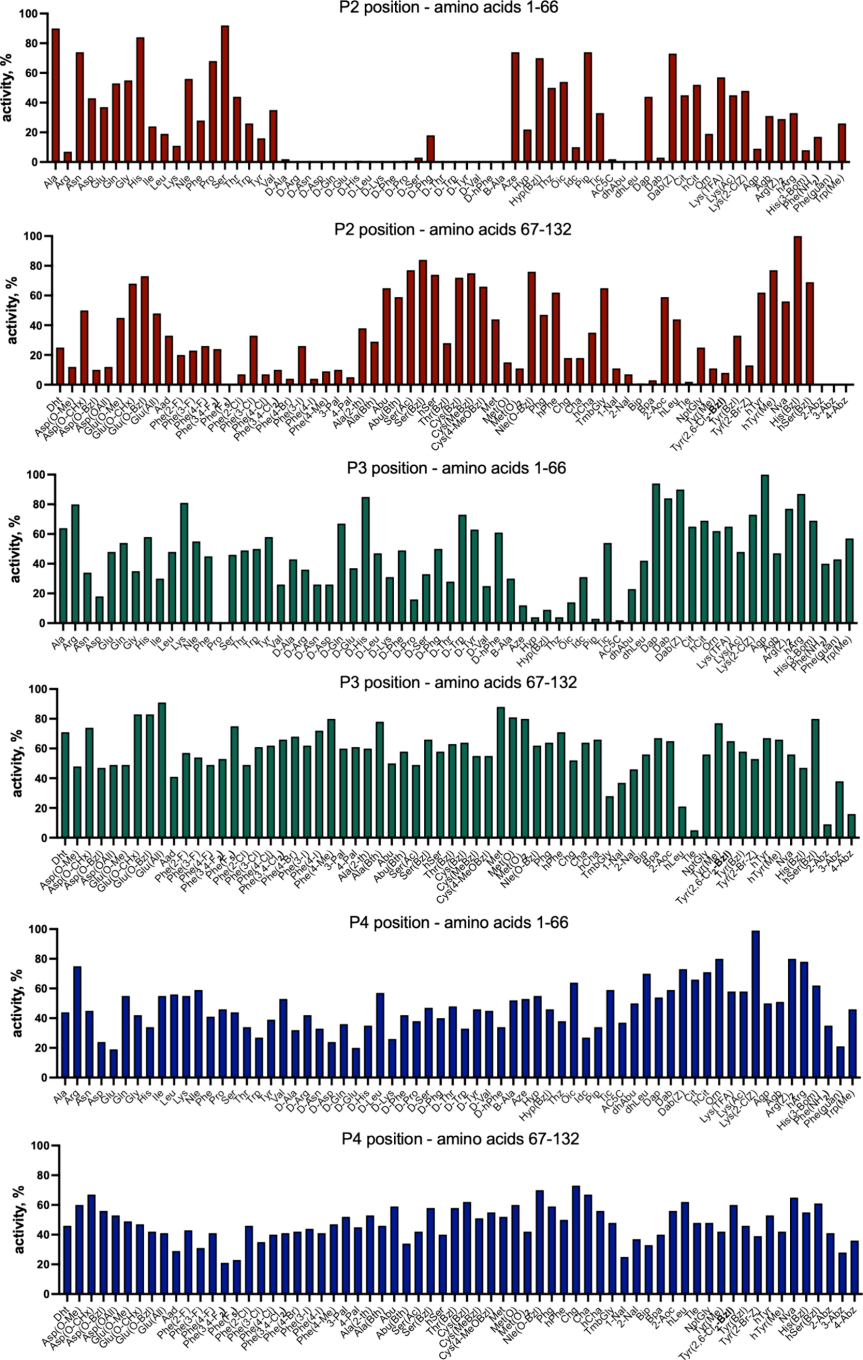
Substrate specificity profile of trypsin determined using the HyCoSuL peptide library. An Ac-P4-P3-P2-Arg-ACC hybrid combinatorial substrate library (HyCoSuL) was used to investigate trypsin substrate preferences at the P2, P3, and P4 positions. Enzymatic activity was measured based on fluorescence release following trypsin cleavage at the P1-Arg residue. The data represent the relative cleavage efficiency for each amino acid, normalized to the most efficiently recognized residue at each position: His­(Bzl) for P2, Agp for P3, and Lys­(2ClZ) for P4. The *x*-axis indicates the natural and unnatural amino acids tested, whereas the *y*-axis shows the relative activity as a percentage of the maximal signal for each position.

### PAD Substrate Specificity

Recent studies have provided important insights into the sequence specificity of peptidylarginine deiminases (PADs), particularly PAD2 and PAD4, using peptide libraries and proteomic profiling.^15,16^ These enzymes do not act indiscriminately on all arginine residues but rather recognize specific sequence motifs in the local environment surrounding the target site. In a large-scale citrullinome study, Assohou-Luty et al. identified key patterns among more than 300 PAD2- and PAD4-catalyzed citrullination sites.^15^ PAD2 displayed a preference for glycine at the +1 position (P1’ according to Schechter and Berger nomenclature) and tyrosine at +3 (P3′), indicating a preference for small or flexible residues near the citrullination site. In contrast, PAD4 exhibited more distinct and narrow preference. One of the most prominent features of PAD4 recognition is the strong enrichment of aspartic acid (D) at the −1 position (P2), a negatively charged residue that likely forms favorable electrostatic interactions with the active site. PAD4 also prefers glycine and aspartic acid at the +1 position (P1’), further emphasizing its selectivity for sequences that are both small and polar or acidic. These findings underscore the importance of both upstream and downstream residues in dictating substrate recognition by PADs. Complementary biochemical studies by Knuckley et al. explored the catalytic behavior of PAD1, PAD3, and PAD4 using synthetic arginine-containing substrates and peptides.^16^ Their work confirmed isozyme-specific differences in catalytic efficiency and substrate recognition, with PAD3 displaying markedly reduced activity toward standard substrates compared with PAD1 and PAD4. Together, these studies highlight that PAD substrate specificity is governed not only by the presence of arginine but also by the surrounding amino acid context, especially the residues at the −1 (P2), + 1 (P1’), and +3 (P3′) positions. The presence of aspartic acid at the −1 position is particularly favorable for PAD4 activity, whereas glycine and tyrosine at downstream positions enhance recognition by PAD2.

To further dissect PADs’ peptide preferences of PADs, we profiled PAD1, PAD2, PAD3, and PAD4 using a HyCoSuL-derived tetrapeptide library containing only natural amino acids (Figure 3A). HyCoSuL enabled the profiling of PAD substrate preferences at the P4, P3, and P2 positions, with a fixed P1 arginine as the citrullination site. Our data revealed that PAD1 and PAD2 share several similarities in terms of substrate recognition. At the P2 position, both enzymes preferentially recognized small or polar residues, such as glycine, lysine, serine, and threonine. In contrast, PAD3 and PAD4 exhibited different specificity patterns. These two enzymes showed overlapping preferences, particularly favoring aspartic acid and serine at the P2 position, suggesting distinct recognition motifs compared with those of PAD1 and PAD2. Based on these observations, PADs can be broadly classified into two specificity groups: PAD1 and PAD2 form one group, and PAD3 and PAD4 form another group, based on their shared substrate profiles. At the P3 position, PAD1 and PAD2 exhibited broad specificity, with notable preferences for glutamine and serine. PAD3 and PAD4 exhibited narrower specificities at this position. Interestingly, arginine emerged as the most preferred amino acid for PAD3 and PAD4 at P3, although subtle differences in the recognition patterns between the two enzymes were still evident. In terms of P4 specificity, PAD2 demonstrated the broadest substrate tolerance among all isozymes, recognizing a wide variety of residues. PAD1 and PAD3 displayed moderate overlap in their preferences at P4, whereas PAD4 had a more distinct profile, strongly favoring arginine, glycine, and tryptophan. In our previous study,^31^ we demonstrated that PAD activity can be modulated not only by calcium ions but also by heparin (in the presence of low concentrations of calcium ions). To evaluate whether different activators influence substrate specificity, we profiled PAD4 using the same tetrapeptide libraries under two activation conditions: high calcium concentration (5 mM) and low calcium concentration (0.1 mM) supplemented with heparin (Figure 3B). The results showed that the PAD4 substrate preferences at the P2, P3, and P4 positions remained largely unchanged under both conditions. This indicates that although calcium and heparin can modulate PAD activity levels, they do not significantly alter the intrinsic substrate specificity of PAD4.

**Figure 3:**
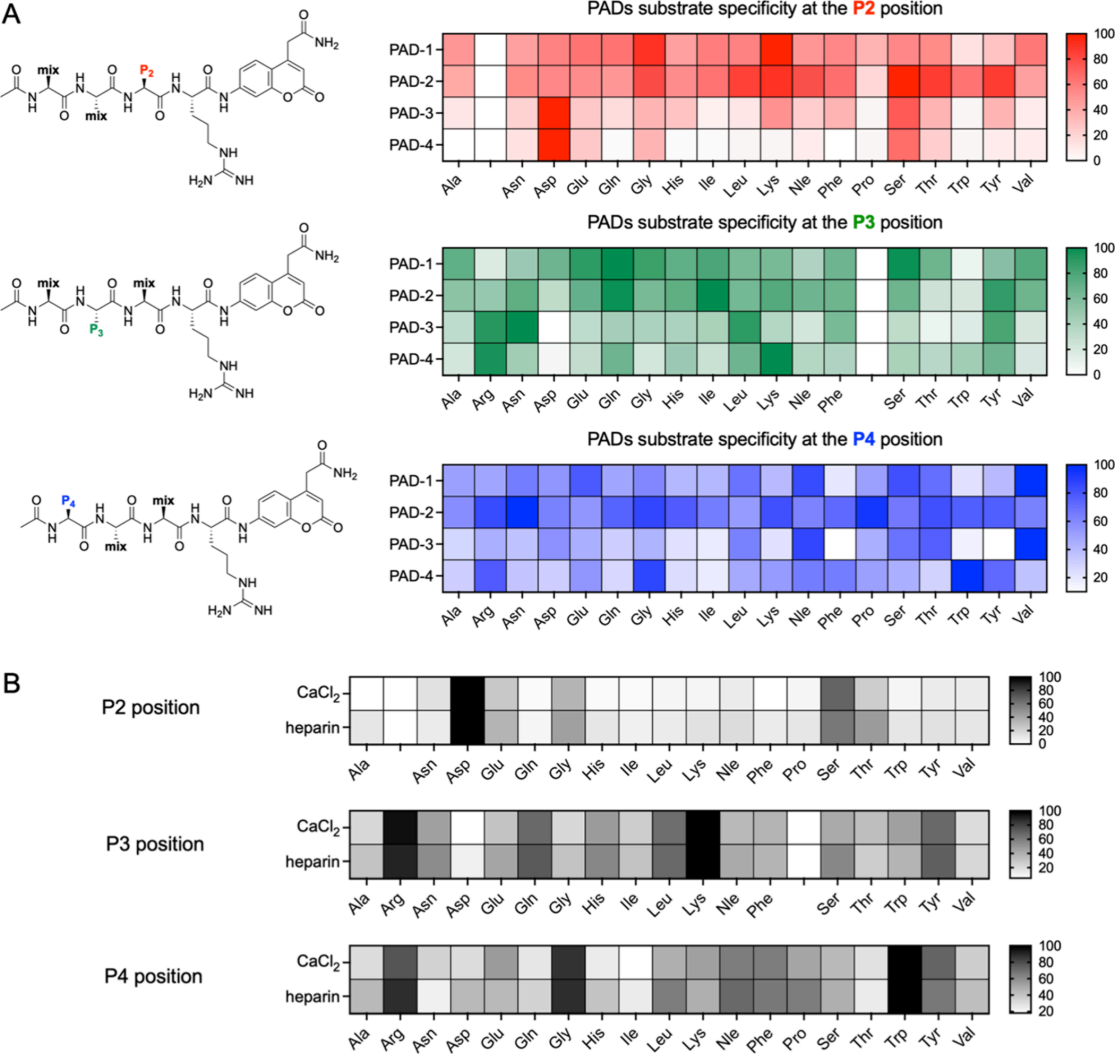
PAD substrate specificity at the P4–P2 positions toward natural amino acids.(A) Substrate specificity profiles of PAD1, PAD2, PAD3, and PAD4 were evaluated at P2, P3, and P4 positions using the Ac-P4-P3-P2-Arg-ACC HyCoSuL library, where the P1 position was fixed as arginine. The relative enzymatic preferences for natural amino acids at each position were visualized as color-coded heat maps, with the most intense color representing 100% relative activity, normalized to the most efficiently cleaved substrate for each PAD isoform. (B) PAD4 substrate specificity at P4–P2 was further assessed under two distinct conditions: high CaCl_2_ concentration (maximally activating) and low CaCl_2_ concentration supplemented with heparin (allosteric modulator). The results are displayed as grayscale heat maps, where black indicates the highest relative activity for a given amino acid at each position. Arginine at the P2 position was excluded from the analysis because the Ac-P4-P3-Arg-Arg-ACC substrate underwent rapid citrullination at P2. The resulting product (Ac-P4-P3-Cit-Arg-ACC) was more efficiently recognized by trypsin than the original substrate was. Additionally, proline at the P3 position was excluded because trypsin does not tolerate this residue at that site.

### PAD2 and PAD4 Specificity toward Unnatural Amino Acids

To enable the development of PAD isozyme-selective substrates, we expanded our substrate profiling efforts beyond natural amino acids by employing HyCoSuL, which contains over 100 unnatural amino acid derivatives. This allowed us to dissect and compare the substrate specificities of PAD2 and PAD4 at high resolution (Figure 4,Figure S1, S2). To our knowledge, this is the first example of applying a HyCoSuL library, traditionally used for protease specificity profiling, to a nonprotease enzyme family. At the P2 position, PAD2 exhibited notably broad substrate tolerance. In addition to efficiently recognizing nearly all natural amino acids, PAD2 accepts a wide variety of unnatural residues with diverse structural and physicochemical properties. These include bulky hydrophobic amino acids, polar uncharged residues, and charged analogs. This finding underscores the high plasticity of PAD2’s substrate-binding site at P2, making it an attractive target for the design of selective fluorogenic or activity-based probes. In contrast, PAD4 displayed a significantly narrower specificity at the P2 position. Aspartic acid emerged as the dominant recognized residue, consistently yielding the highest activity among natural and unnatural amino acids. Serine and threonine were also tolerated, reflecting a preference for small, polar, or slightly branched side chains in the substrate. Among the unnatural amino acids tested, only a few were recognized by PAD4 to a measurable extent, but none surpassed or equaled the efficiency observed for aspartic acid. These results demonstrate that PAD4 is highly selective at the P2 position, in contrast to the broader substrate flexibility of PAD2. Analysis at the P3 position revealed that both PAD2 and PAD4 displayed broader substrate recognition than P2. However, notable distinctions remained between the two enzymes. PAD4 preferentially recognizes certain unnatural amino acids, such as Dap (2,3-diaminopropionic acid), suggesting specific interactions within the P3-binding pocket. PAD2, in contrast, demonstrated a bias toward more hydrophobic residues, indicating differences in surface hydrophobicity and steric accommodation at the binding site. These positional preferences provide useful discriminatory features for designing isozyme-selective substrates and inhibitors. At the P4 position, both enzymes showed a generally broad specificity. Nevertheless, screening revealed distinct trends. PAD2 displayed a somewhat higher tolerance for D-amino acids than PAD4, which could be used to selectively enhance PAD2 reactivity. Conversely, PAD4 was more responsive to bulky, hydrophobic amino acid residues that were poorly accepted by PAD2, suggesting key differences in the shape and electrostatic characteristics of the P4 subsite. In summary, HyCoSuL-based profiling of PAD2 and PAD4 with unnatural amino acids revealed differences in substrate selectivity across all tested positions. PAD2 exhibits broader and more permissive substrate specificity, especially at P2, whereas PAD4 demonstrates stricter preferences, particularly favoring acidic or small polar residues.

**Figure 4:**
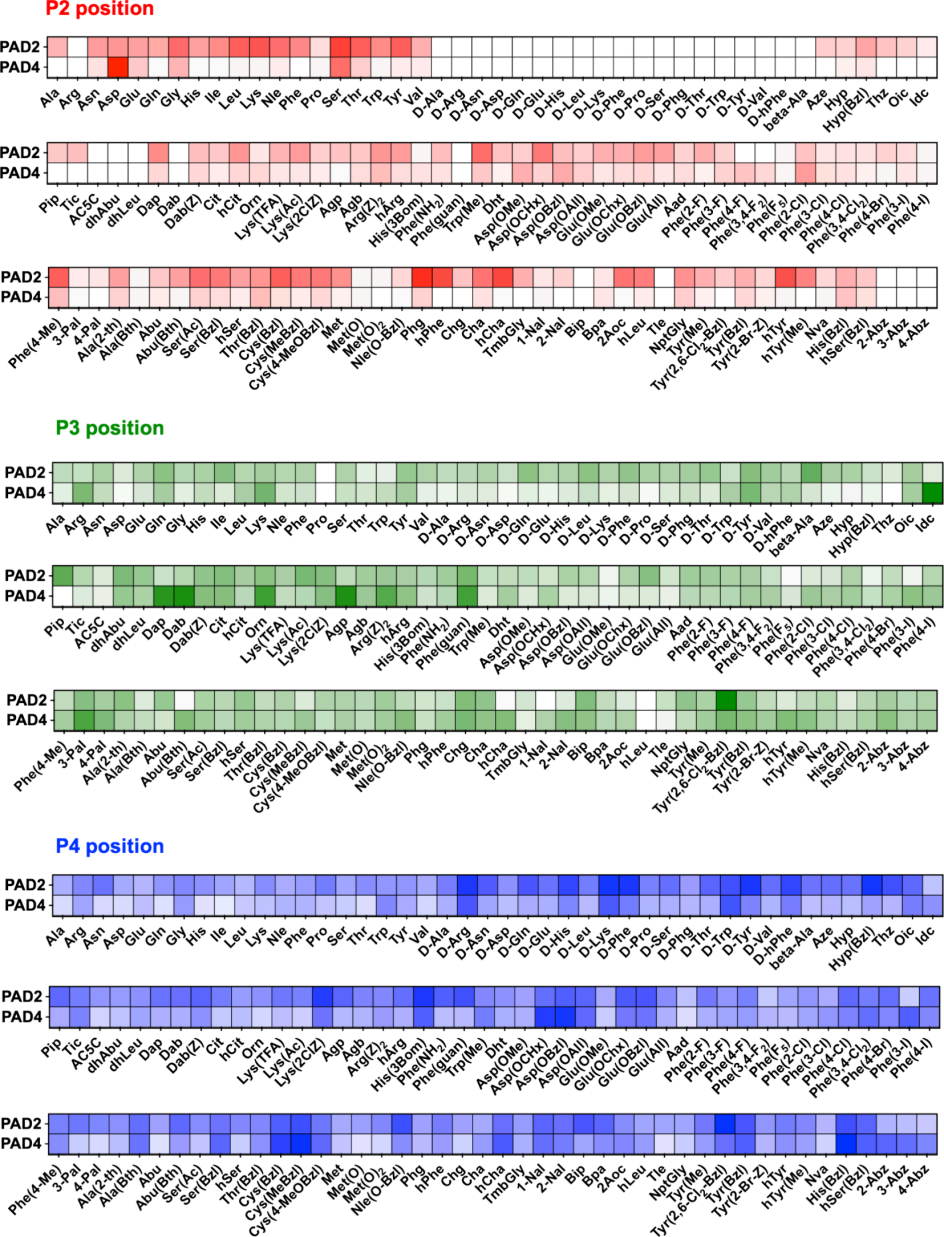
Broad substrate specificity of PAD2 and PAD4 profiled using HyCoSuL peptide libraries. The substrate preferences of PAD2 and PAD4 were analyzed at P2, P3, and P4 using sublibraries derived from the Ac–P4–P3-P2-Arg-ACC HyCoSuL platform. Each sublibrary was designed to fix one position while keeping the other two randomized: Ac-Mix-Mix-P2-Arg-ACC for P2, Ac-Mix-P3-Mix-Arg-ACC for P3, and Ac-P4-Mix-Mix-Arg-ACC for P4. Each sublibrary contained 19 natural amino acids (except Cys) and 113 unnatural amino acids. Enzymatic preferences were visualized as heat maps, where the most intense color indicated 100% relative citrullination activity, and all other values were scaled accordingly. The data demonstrate the broad substrate tolerance and selectivity of PAD2 and PAD4 across the extended substrate positions P4 to P2, presented as relative citrullination rates ranging from 0 to 100%.

### Kinetic Analysis of PAD2 and PAD4 Selective Substrates

After determining the substrate specificity profiles of PAD2 and PAD4 using the HyCoSuL libraries, we sought to apply this information to design selective ACC-labeled substrates capable of distinguishing between these two enzymes. Based on our profiling data, we synthesized a focused panel of substrates: eight candidates tailored for PAD4 and nine for PAD2 (Figure 5A,Table S1, S2, Figure S3–S19). For PAD4-selective substrates, we introduced bulky hydrophobic residues at the P4 position, such as His­(Bzl), Asp­(Bzl), hCha, and Phe derivatives. At the P3 position, we incorporated residues including Leu, Arg, DTyr, and hCha based on their ability to enhance selectivity toward PAD4 over PAD2. Critically, aspartic acid was used at the P2 position in all PAD4 substrates, as it emerged from our screening as the most selective amino acid for PAD4. For PAD2-selective substrates, we used either bulky aliphatic amino acids, such as Nle­(OBzl) at P4, D-amino acids, such as DTyr, or small and polar residues, such as Asn and positively charged His. At the P3 position, we introduced a diverse set of residues, including branched aliphatic amino acids (Ile and Val), Gln, and Tyr. The P2 position in PAD2 substrates predominantly features bulky or hydrophobic residues, such as Tyr, hPhe, hCha, and Leu. Kinetic analysis using recombinant PAD enzymes confirmed that PAD4 efficiently citrullinated all eight PAD4-specific substrates, even at low concentrations (as low as 37.5 nM), and to a lesser extent for (2)-NH-16 and (2)-NH-18 substrates (Figure 5B). PAD2 was found to be less catalytically active under the assay conditions; however, it effectively citrullinated its designated substrates at higher enzyme concentrations (150–300 nM). Importantly, PAD4 substrates were either poorly recognized or not citrullinated by PAD2, with the exception of two substrates, (2)-NH-11 and (2)-NH-13, which showed some degree of cross-reactivity. It is more challenging to design PAD2 substrates that exhibit high selectivity for PAD4. Nevertheless, several candidates demonstrated acceptable selectivity, with PAD2 requiring lower concentrations to fully citrullinate the substrates compared to PAD4. The most selective PAD4 substrates identified were **(4)-NH-2** Asp­(Bzl)-DTyr-Asp-Arg, and **(4)-NH-12** Phe­(3I)-3 Pal-Asp-Arg. These sequences were efficiently processed by PAD4 but poorly recognized by PAD2. For PAD2, the most selective substrates were **(2)-NH-7** DTyr-Ala-hPhe-Arg, and **(2)-NH-8** Asn-Val-hCha-Arg (Figure 5C). These substrates exhibited higher reactivity with PAD2 and only minor processing by PAD4, particularly at lower PAD4 concentrations. Overall, these findings demonstrate that the HyCoSuL-guided approach can be successfully applied to engineer PAD-isozyme-selective substrates. The kinetic data validated the feasibility of designing ACC-containing fluorescent substrates with high selectivity for PAD2 or PAD4. This strategy provides a valuable framework for developing future PAD-specific inhibitors or activity-based probes.

**Figure 5:**
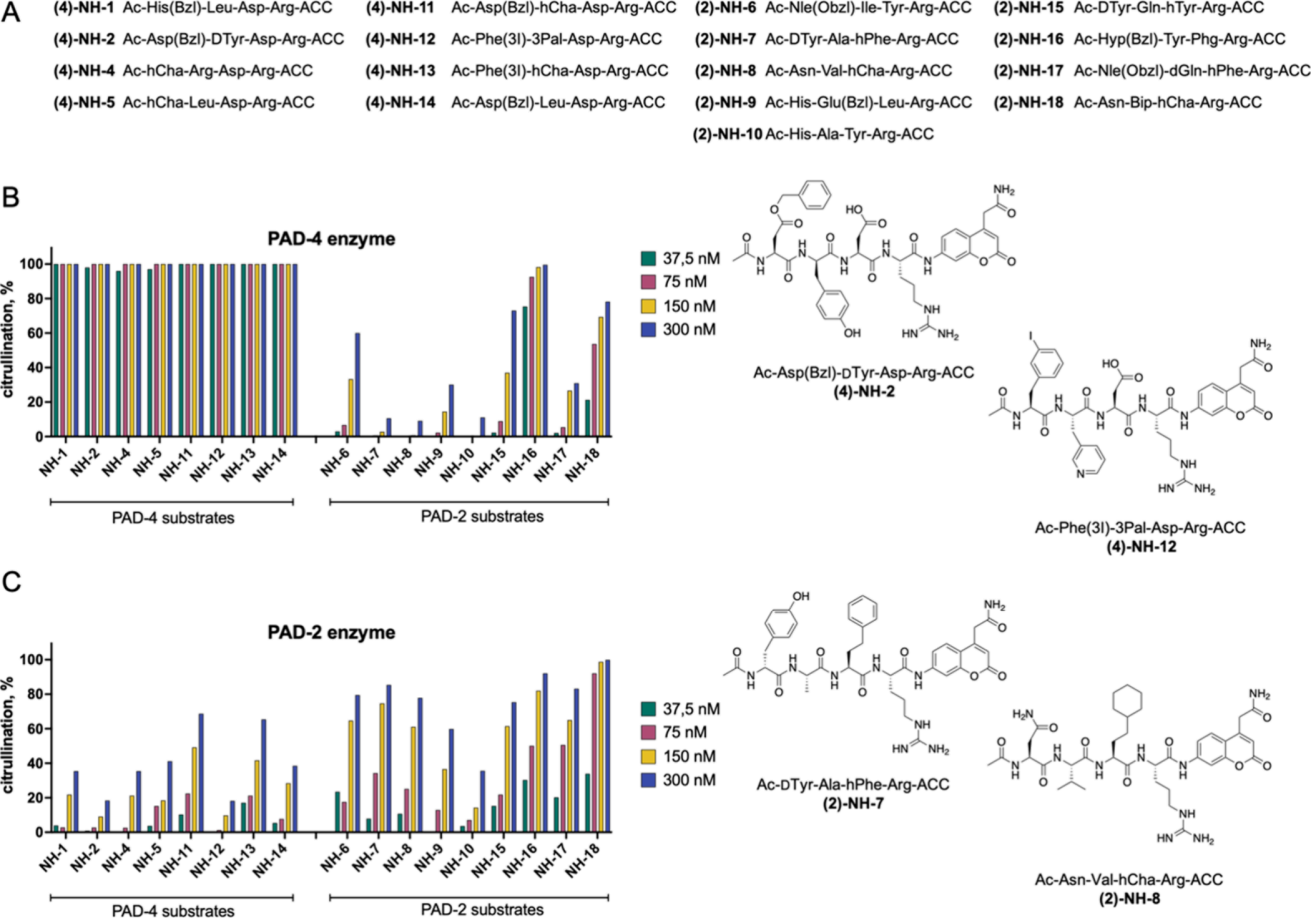
Development of PAD2- and PAD4-selective substrates.(A) HyCoSuL screening data was used to select amino acids for the design of selective tetrapeptide substrates specific to PAD4 (eight substrates, left) and PAD2 (nine substrates, right). All substrates followed the general structure Ac–P4–P3-P2-Arg-ACC. Variability at the P4–P2 positions was employed to confer enzyme selectivity to the substrates. (B) C Specificity analysis of individual substrates toward PAD4 (C) and PAD2 (D) along with most selective substrates. Substrates were incubated with enzymes for 15 min at various concentrations, followed by trypsin addition to assess cleavage of noncitrullinated substrates. The extent of trypsin-mediated fluorescence release reflects the residual noncitrullinated substrate and reveals the substrate preferences of each enzyme. Chemical structures of the most selective substrates identified for PAD2 and PAD4, all of which include unnatural amino acids. The most selective substrates for PAD4 are **(4)-NH-2** Ac-Asp­(Bzl)-DTyr-Asp-Arg-ACC and **(4)-NH-12** Ac-Phe­(3I)-3Pal-Asp-Arg-ACC, while those for PAD2 are **(2)-NH-7** Ac-DTyr-Ala-hPhe-Arg-ACC and **(2)-NH-8** Ac-Asn-Val-hCha-Arg-ACC.

### Analysis of PAD Activity in Cells

PAD2 and PAD4 play critical roles in immune cell function, particularly in monocytes and macrophages, where their activity has been implicated in inflammatory signaling, differentiation, and cell death pathways.^10,40^ Both enzymes are expressed in macrophages under specific activation states, and their expression is regulated during differentiation, such as in THP-1 cells treated with phorbol esters.^41^ PAD4 is well-known for its role in chromatin decondensation through histone citrullination, a key step in NETosis in neutrophils; however, similar mechanisms are increasingly recognized in macrophages, including the formation of macrophage extracellular traps (METs).^42^ In particular, PAD4-dependent citrullination is associated with nuclear decondensation in pro-inflammatory M1 macrophages exposed to NETs. Furthermore, PAD2 and PAD4 contribute to inflammasome activation and pyroptotic cell death.^43^ Both enzymes regulate NLRP3 inflammasome assembly and IL-1β maturation in macrophages, and their inhibition significantly reduces pro-inflammatory cytokine release. Importantly, the activation of PAD enzymes correlates with elevated intracellular calcium concentrations, which are often associated with cellular stress or death signals. In macrophages, this activation leads to the citrullination of cytoskeletal proteins, such as vimentin, a process linked to cytoskeletal remodeling and apoptosis.^44^ Collectively, these findings highlight the central role of PADs in citrullination-dependent signaling and post-translational modification, as well as in modulating macrophage responses during immune activation, differentiation, and regulated cell death. These mechanisms form the foundation for the experimental evaluation of PAD activity in differentiated THP-1 macrophages and primary immune cell lysates. In our experiments, we focused on the regulation of vimentin expression during phorbol 12-myristate 13-acetate (PMA)-induced monocyte-to-macrophage differentiation in THP-1 cells and examined the involvement of PAD4, calpain-1, and caspase-3 in this process. PMA-differentiated THP-1 macrophages serve as a foundation for further inflammasome activation, and our goal was to assess how changes in vimentin levels influence this capability. We observed that increasing concentrations of PMA led to a dose-dependent upregulation of vimentin, while concurrently downregulating key inflammasome components, such as GSDMD and NLRP3 (Figure 6A). Interestingly, similar regulatory effects were observed when THP-1 cells were pretreated with inhibitors of PAD4 (PADi, Cl-amidine), caspase-3 (C3i, Z-DEVD-fmk) and calpain-1 (Calp, calpeptin) (Figure 6B). These findings suggest a functional interplay between these enzymes and inflammasome-related proteins during macrophage differentiation. This is consistent with recent studies indicating that PADs, particularly PAD2 and PAD4, play a regulatory role in macrophage polarization and inflammasome activation.^40^ We tested the ability of PAD4 to citrullinate vimentin by incubating recombinant vimentin with recombinant PAD4, which resulted in efficient citrullination (Figure 6C). When native or citrullinated vimentin was treated with caspase-3, both forms were cleaved at the same sites and exhibited similar kinetics. Notably, the cleavage of citrullinated vimentin by calpain-1 was delayed compared to that of the nonmodified form. Fifteen minutes after the addition of calpain-1, the full-length native vimentin had almost completely disappeared, whereas the full-length citrullinated form remained detectable even after 30 min (Figure 6D). Consequently, the 28 kDa cleavage fragment of the citrullinated protein was less prominent, and the smallest fragment of approximately 4 kDa was absent, in contrast to the cleavage pattern observed for nonmodified vimentin. This difference can be attributed to the substrate specificity of calpain-1, which prefers arginine residues in the P1 position. During citrullination, arginine is converted to citrulline, which appears to be a less favorable substrate for calpain-1.^45^ To directly measure PAD activity in living cells, we employed a trypsin-based kinetic assay using PAD2- and PAD4-selective substrates (Figure 6E). In THP-1 cells differentiated with PMA alone, PAD activity was low but detectable. However, upon subsequent stimulation with LPS, PAD activity significantly increased, particularly for PAD2, consistent with previous reports that PAD2 is the most abundant isoform in activated THP-1 macrophages.^40^ Although PAD4 activity was also measurable, it was lower, indicating that LPS stimulation selectively enhanced PAD2-mediated citrullination in this model. These findings support the emerging view that PAD activity in immune cells is dynamically regulated by differentiation and inflammatory stimuli and that PAD2 plays a critical role in monocyte-derived macrophages. Our data complement and extend recent observations that PAD2 promotes M1 polarization and serves as a key modulator of macrophage phenotype and function.

**Figure 6:**
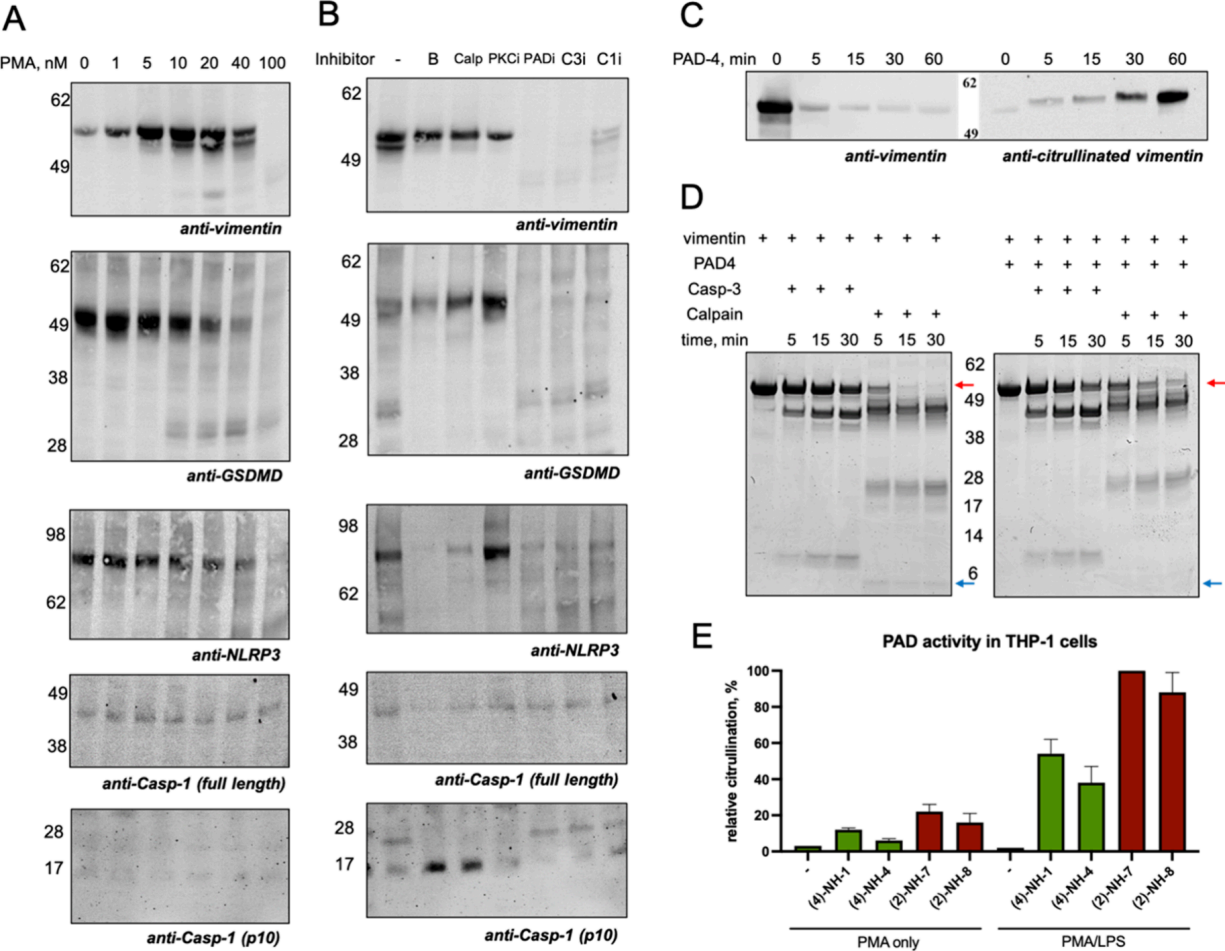
Analysis of PAD-mediated citrullination during pyroptosis. (A) THP-1 cells were differentiated using PMA at increasing concentrations, followed by immunoblotting analysis. High concentrations of PMA induced vimentin upregulation and proteolytic processing, while simultaneously leading to the downregulation of GSDMD and NLRP3 protein levels. (B) THP-1 cells were pretreated with various inhibitors prior to differentiation with 40 nM PMA. In the presence of the calcium chelator BAPTA (B) or the calpain inhibitor calpeptin (Calp), caspase-1 was processed into its active 10 kDa fragment, followed by the cleavage of GSDMD. In contrast, pretreatment with either the PAD inhibitor (PADi, Cl-amidine) or caspase inhibitors (C3i, Z-DEVD-fmk for caspase-3 and C1i, VX-765 for caspase-1) prevented the formation of caspase-1 p10 and led to a marked downregulation of both vimentin and GSDMD. (C) PAD4 rapidly citrullinates vimentin, as demonstrated by immunostaining with antibodies specific for vimentin and its citrullinated forms, revealing early and robust citrullination after stimulation. (D) Distinct cleavage rate of vimentin by calpain-1 were observed when incubated with native versus PAD4-citrullinated vimentin. Citrullination significantly delayed the cleavage of vimentin by calpain-1 and the distribution of cleaved fragments (red/blue arrows). (E) PAD enzymatic activity was measured in PMA-only and PMA/LPS-stimulated THP-1 cells using four fluorogenic tetrapeptide substrates. Activity was normalized using a previously established trypsin-coupled assay and is presented on the *y*-axis as relative fluorescence intensity.

### PAD-Mediated Citrullination of Vimentin Diminishes Its Function as a Damage-Associated Molecular Pattern (DAMP)

DAMPs are molecules released in response to cellular stress and tissue injury. These endogenous danger signals can activate the innate immune system and trigger potent inflammatory responses during noninfectious inflammation.^46^ As citrullination of various proteins has been shown to modify their immunogenicity and contribute to the pathogenesis of inflammatory diseases (e.g., rheumatoid arthritis and ulcerative colitis), this study examined the impact of citrullination on the extracellular activity of vimentin as a DAMP.^47,48^ We first incubated PMA/LPS-treated THP-1 cells with increasing amounts of vimentin or PAD4-mediated citrullinated vimentin (cit-vimentin) to evaluate their potential extracellular activity as DAMPs Our results showed that a high concentration of vimentin (10 μg), but not cit-vimentin, induced cell membrane disruption, as measured by lactate dehydrogenase (LDH) release (Figure 7A). Next, we tested the effects of vimentin and cit-vimentin fragments generated by proteolytic cleavage by caspase-1 or caspase-3. However, no significant cytotoxic effects were observed under any of these conditions, except when cells were treated with intact vimentin or nigericin, which served as a positive control known to induce pyroptosis (Figure 7B**).** These findings suggest that proteolytic processing of vimentin abolishes its DAMP-like activities. Importantly, although both vimentin and nigericin triggered cell death, they appeared to do so through distinct mechanisms. Nigericin, a classical pyroptosis inducer, activated caspase-1, leading to the cleavage of gasdermin D (GSDMD) into its p31 fragment and the activation of pro-interleukin-1β (Figure 7 **C, D**). In contrast, treatment with vimentin or cit-vimentin resulted in the processing of GSDMD into the p45 fragment, which is characteristic of caspase-3-mediated cleavage, with no cleavage of pro-IL-1β (Figure 7 **C, D**). Collectively, these data indicate that extracellular vimentin functions as a DAMP by activating caspase-3 and inducing apoptotic rather than pyroptotic cell death.

**Figure 7:**
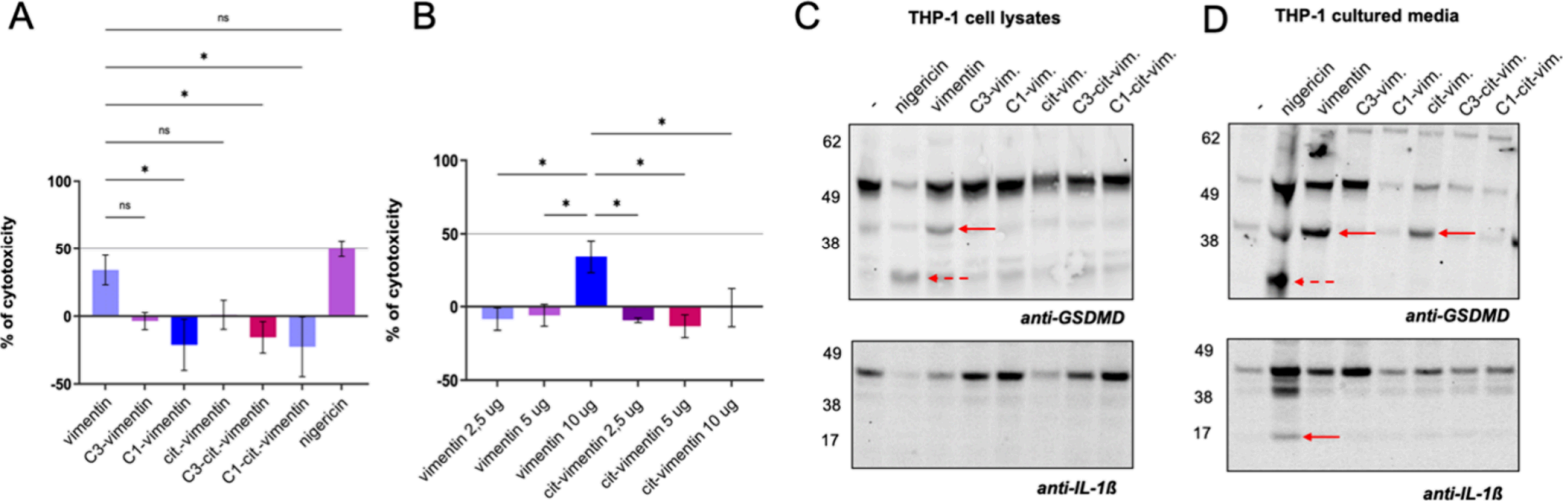
Analysis of extracellular vimentin and citrullinated vimentin as damage-associated molecular patterns. (A) The addition of extracellular vimentin to PMA/LPS-stimulated THP-1 cells resulted in LDH release, indicating membrane disruption and subsequent cell death. (B) The cytotoxic effects of vimentin, citrullinated vimentin, and their cleavage products generated by caspase-1 and caspase-3 were evaluated. Nigericin stimulation served as a positive control for cell death. (C, D) Western blot analysis revealed that exposure of the cells to vimentin and its cleavage fragments led to the formation of the p43 GSDMD fragment (red arrows, C, D; upper panel), which inactivated GSDMD and thereby prevented pyroptosis. This was further confirmed by the absence of the active form of IL-1β (C, D bottom panel). Cleaved IL-1β was detected only in the positive control for pyroptotic cell death (nigericin; D, bottom panel), which also generated the p31 kDa GSDMD fragment cleaved by caspase-1 (C, D; red dashed arrows). Notably, the effects of vimentin stimulation were diminished when cells were treated with citrullinated vimentin (cit-vim) and completely abolished upon exposure to the cleavage products of both vimentin forms generated by caspase-3 and calpain-1 (C3-, C1-vimentin and C3-, C1-cit-vimentin). *p < 0.05.

## Discussion

Our substrate specificity profiling of PADs with libraries containing natural amino acids revealed a clear contrast in P2 residue preferences among PAD isozymes, as PAD1 and PAD2 accommodate a broad range of residues at the P2 position, whereas PAD3 and PAD4 show a strong preference for acidic residues (especially Asp, and to a lesser extent Ser/Thr). This pattern can be partially rationalized by differences in the active-site architecture and electrostatic environment of the enzymes. Funabashi et al. compared PAD1–4 structures and highlighted isozyme-specific differences near the active site (e.g., Arg374 in PAD4 vs Gly374 in PAD3) that alter pocket charge and shape, which could influence how residues adjacent to the target arginine (e.g., at P2 position) are accommodated.^49^ Therefore, in PAD4, Arg374 can directly engage substrate or inhibitor molecules and likely serves as a positively charged anchor that preferentially stabilizes acidic side chains at P2. In contrast, PAD3 Gly374 cannot provide such an interaction, resulting in a pocket that, while larger, lacks the cationic anchor. This may seem counterintuitive given PAD3 preference for Asp at P2, but it suggests that other active-site elements (e.g., neighboring residues like Arg372 or overall electrostatic context) still favor binding of polar residues in that spacious pocket. Meanwhile, the more permissive pockets of PAD1 and PAD2, which do not enforce a strict electrostatic or steric bias, can accommodate a variety of P2 side chains.

HyCoSuL profiling of PAD2 and PAD4 specificity with a broad range of unnatural amino acids revealed further differences in substrate recognition. PAD4 exhibited a markedly restricted substrate preference, most notably favoring aspartate at the P2 position adjacent to the citrullination site, whereas PAD2 accommodated a broader range of residues at this position. This finding aligns with earlier reports that human PAD4 has a more selective sequence motif than PAD2. Such distinct specificities likely reflect structural differences in the enzyme active sites and highlight that each PAD isoform targets a unique subset of proteins within the cell.^15^ By exploiting these preferences, we developed fluorogenic peptide substrates incorporating unnatural amino acids that are selectively deiminated by PAD2 or PAD4. The use of unnatural residues expanded the chemical diversity of the library, allowing the identification of isoform-specific substrates that would not be obtainable from natural amino acids alone. These optimized substrates showed minor cross-reactivity, enabling reliable discrimination between PAD2 and PAD4 activities in complex biological samples. The validation of isoform-selective substrates in biochemical assays confirmed that the introduced unnatural amino acids conferred high selectivity without compromising catalytic efficiency. In lysate-based **trypsin-coupled activity assays**, the PAD2-selective peptide and PAD4-selective peptides reported enzyme activity even in the presence of the other isoform. This allowed us to measure the contribution of each PAD in cell-derived samples, providing insights into their roles in immune cell function. In differentiated THP-1 macrophage lysates, PAD2 activity predominated, consistent with the known upregulation of PAD2 during monocyte-to-macrophage differentiation.^40^ This suggests that inflammatory signaling can acutely elevate PAD2 activity, potentially through calcium mobilization or upregulation of PAD2 expression, as observed with calcium ionophore treatment. Our data suggest that PAD2 is the major contributor to citrullination events in activated macrophages, whereas PAD4 plays a lesser role. This distinction is important because PAD4 is often linked to nuclear events (e.g., chromatin deimination during neutrophil NET formation), whereas PAD2 may regulate cytosolic or cytoskeletal targets in macrophages.

The present study demonstrated that PAD-driven citrullination of vimentin does not influence caspase-3 proteolysis and cleavage. This result aligns with the known substrate specificity of caspase-3, which has a high preference for the Asp-Glu-Val-Asp sequence.^50^ Notably, these amino acids are not substrates for PAD enzymes, suggesting that the sites of citrullination in vimentin do not interfere with caspase-3 cleavage sites. Our findings contrast with those of Jang et al., who reported that citrullination impedes caspase-3-mediated cleavage.^51^ However, in that study, cleavage fragments were detected using anticitrullinated vimentin antibodies, which have been shown to exhibit reduced specificity for cleaved vimentin fragments. This limitation was also observed in our investigation, where attempts to detect caspase-3 cleavage products using anticitrullinated vimentin antibodies were unsuccessful. In contrast, Coomassie staining, which detects all proteins without specificity, revealed no difference in the processing of native versus PAD-modified vimentin by caspase-3. Discrepancies may also arise due to the use of different PAD enzymes. In the present study, PAD4 was employed, while the aforementioned authors used PAD2. Conversely, our observation that PAD4-citrullinated vimentin is less susceptible to calpain-1 cleavage supports the conclusion that substrate specificity is critical. As demonstrated previously, arginine, which is converted to citrulline during PAD4 activity, is also the preferred amino acid in the cleavage sequence for calpain.^45,52^ Therefore, it is likely that citrullination sites interfere with calpain-1-mediated processing of vimentin. Citrullination of vimentin has been shown to cause intermediate filament disassembly,^53^ and our results extend this concept by demonstrating that citrullination also influences vimentin cleavage. As demonstrated by other researchers, a variety of proteins exhibit resistance to proteolytic processing by enzymes, such as thrombin, plasmin, and proteinase K, following citrullination, underscoring the significance of this phenomenon.^54–56^ Furthermore, we found that vimentin citrullination diminished its activity as a damage-associated molecular pattern in the human monocytic leukemia cell line THP-1. In contrast to this observation, other authors have recently reported an enhancement of extracellular vimentin DAMP activity toward neutrophils following citrullination (41). Moreover, our observations indicate that extracellular vimentin does not exhibit pro-inflammatory activity in THP-1 cells, whereas the aforementioned studies demonstrated that both native and citrullinated forms of this intermediate filament protein possess pro-inflammatory properties in neutrophils. This divergence highlights the complexity of the interplay between PAD activity and the modulation of innate immune signaling via cytoskeletal DAMPs, suggesting a dynamic and multifaceted network with context-dependent effects.

## Conclusions

Our findings have important implications for the chemical biology of citrullination. The marked preference of PAD4 for particular motifs (e.g., an acidic residue at P2) versus the promiscuity of PAD2 suggests that it is feasible to design highly selective inhibitors or activity-based probes targeting one PAD isoform over another. Indeed, previous studies have shown that understanding PAD substrate preferences can drive inhibitor development, as exemplified by the design of PAD-selective inhibitors guided by substrate profiling. By defining consensus recognition sequences for PAD2 and PAD4, our study provides a foundation for the future development of selective PAD inhibitors or peptidyl probes. Given that dysregulated citrullination is linked to numerous diseases (from rheumatoid arthritis to cancer), tools that can distinguish PAD isoform functions will be invaluable for dissecting their individual roles in pathogenesis. We hypothesize that the substrate-based approach demonstrated here will facilitate the creation of new PAD-selective reagents and therapeutics, ultimately advancing our ability to monitor and modulate protein citrullination in biological systems. This selective substrate strategy complements ongoing efforts to develop small-molecule PAD inhibitors, and together, these tools move us closer to precise control over PAD activity for research and potential clinical intervention.

## Supplementary Material



## Notes

HyCoSuL substrate specificity profiles at P4–P2 positions for PAD4 and PAD2 (Figures S1 and S2); chemical structure of ACC-containing peptide substrates (Table S1); LC-MS data for all substrates (Table S2, Figures S3–S19) (PDF)

## Notes

PAD1: Q9ULC6, PAD2: Q9Y2J8, PAD3: Q9ULW8, PAD4: Q9UM07, trypsin: P07477, calpain-1: P07384, caspase-1: P29466, caspase-3: P42574.
