# Supplementary material for: Isoform-Selective PAD2/PAD4 Substrates with Unnatural Amino Acids Enable Cellular Peptidylarginine Deiminase Activity Profiling and Reveal Vimentin Citrullination Effects in Macrophages
Source: Biochemistry. 2025 Sep 25;64(19):4105–20. doi: 10.1021/acs.biochem.5c00391 (PMC12509316; doi:10.1021/acs.biochem.5c00391)
Supplement: Supplementary file 1 [file bi5c00391_si_001.pdf]

SUPPORTING INFORMATION

Isoform-selective PAD2/PAD4 substrates with unnatural amino acids enable cellular PAD activity profiling and reveal vimentin citrullination effects in macrophages

Oliwia Gorzeń[1#], Agata Mikołajczyk-Martinez[1,2#,] Abdulla Al. Mamun[1], Natalia Horbach[1], Olha Severynovska[1], Grzegorz Bereta[3], Ewa Bielecka[3], Piotr Mydel[4,5], Marcin Drąg[1,6], Tomasz Kantyka[3], Marcin Poręba[1,7,8,*]

[1]Faculty of Chemistry, Wroclaw University of Science and Technology, 50-370, Wroclaw, Poland, [2]Faculty of Veterinary Medicine, Wroclaw University of Environmental and Life Sciences, 50-375, Wroclaw, Poland, [3]Jagiellonian University, Malopolska Centre of Biotechnology, 30-387, Krakow, Poland, [4]Faculty of Biochemistry, Biophysics and Biotechnology, Jagiellonian University, 30-387, Krakow, Poland, [5]Broegelmann Research Laboratory, University of Bergen, NO-5020, Bergen, Norway, [6]Centre for Chemical Biology, Institute of Physical Chemistry, Polish Academy of Sciences, 01-224, Warsaw, Poland, [7]Faculty of Medicine, Wroclaw University of Science and Technology, 51-377, Wroclaw, Poland; [8]Lead contact; [#]equal contribution, *correspondence: marcin.poreba@pwr.edu.pl

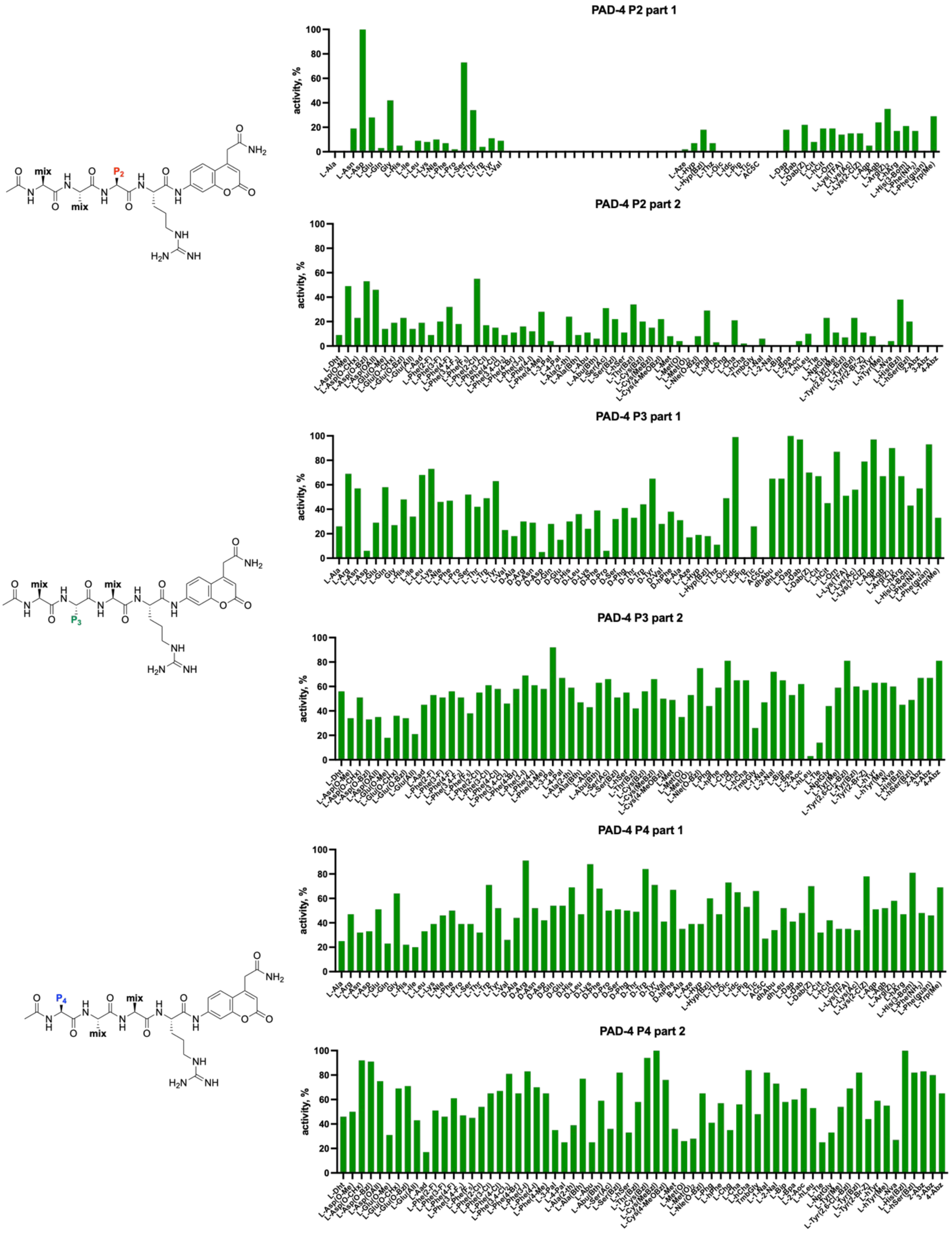

Figure S1 Broad substrate specificity of PAD4 profiled using HyCoSuL peptide libraries. The substrate preferences of PAD4 was analyzed at P2, P3, and P4 using sublibraries derived from the Ac-P4-P3-P2-Arg-ACC HyCoSuL platform. Each sublibrary was designed to fix one position while keeping the other two randomized: Ac-Mix-Mix-P2-Arg-ACC for P2, Ac-Mix-P3-Mix-Arg-ACC for P3, and Ac-P4-Mix-Mix-Arg-ACC for P4. Each sublibrary contained 19 natural amino acids (except Cys) and 113 unnatural amino acids. Enzymatic preferences were visualized as column charts, where the best amino acid was set as 100 percent, and all other values were scaled accordingly.

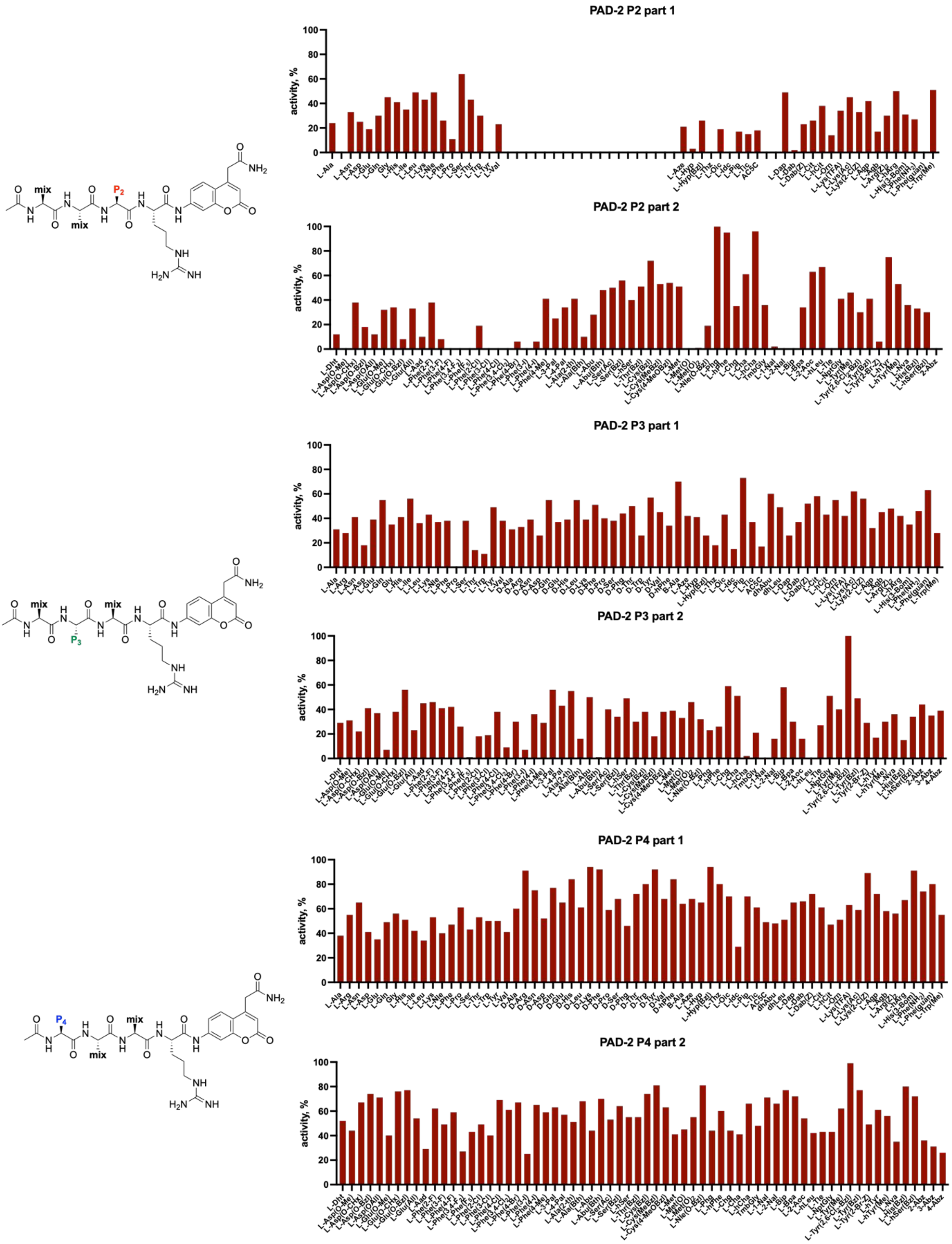

Figure S2 Broad substrate specificity of PAD2 profiled using HyCoSuL peptide libraries. The substrate preferences of PAD2 was analyzed at P2, P3, and P4 using sublibraries derived from the Ac-P4-P3-P2-Arg-ACC HyCoSuL platform. Each sublibrary was designed to fix one position while keeping the other two randomized: Ac-Mix-Mix-P2-Arg-ACC for P2, Ac-Mix-P3-Mix-Arg-ACC for P3, and Ac-P4-Mix-Mix-Arg-ACC for P4. Each sublibrary contained 19 natural amino acids (except Cys) and 113 unnatural amino acids. Enzymatic preferences were visualized as column charts, where the best amino acid was set as 100 percent, and all other values were scaled accordingly.

Table S1 The chemical structures of ACC-based substrates for PAD4 and PAD2 enzymes.

**(4)-NH-1** Ac-His(Bzl)-Leu-Asp-Arg-ACC<br>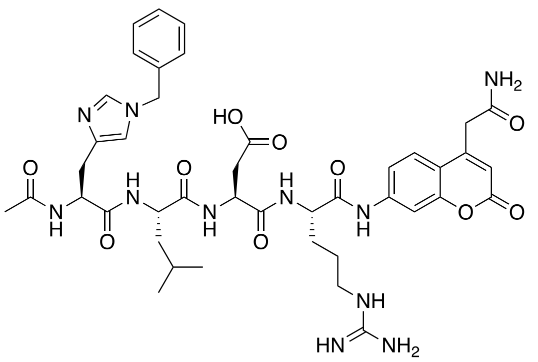	**(4)-NH-2** Ac-Asp(Bzl)-*D*Tyr-Asp-Arg-ACC<br>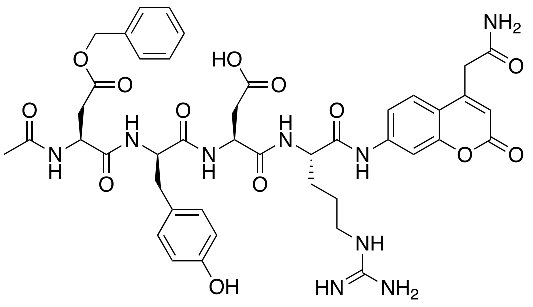
**(4)-NH-4** Ac-hCha-Arg-Asp-Arg-ACC<br>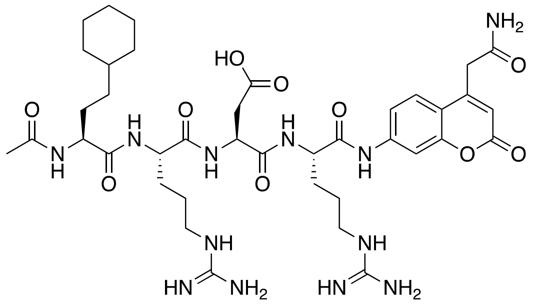	**(4)-NH-5** Ac-hCha-Leu-Asp-Arg-ACC<br>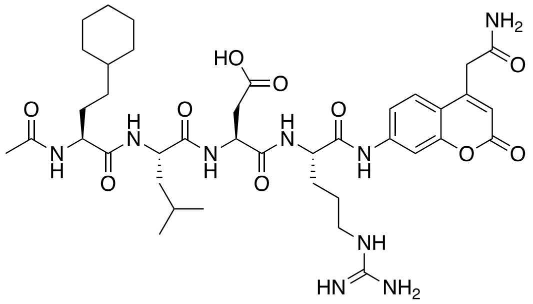
**(4)-NH-11** Ac-Asp(Bzl)-hCha-Asp-Arg-ACC<br>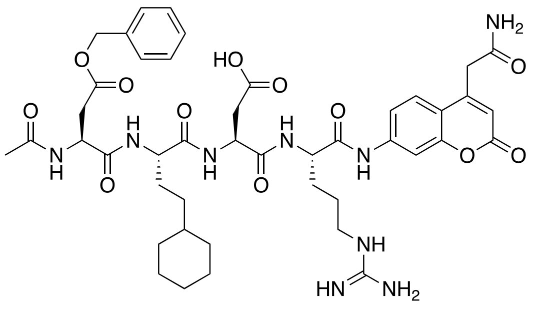	**(4)-NH-12** Ac-Phe(3I)-3Pal-Asp-Arg-ACC<br>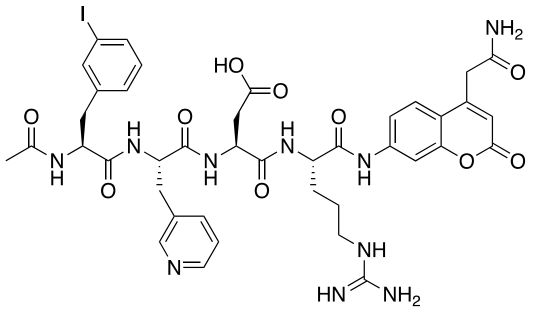
**(4)-NH-13** Ac-Phe(3I)-hCha-Asp-Arg-ACC<br>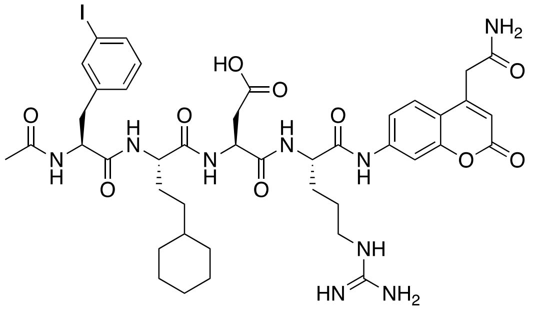	**(4)-NH-14** Ac-Asp(Bzl)-Leu-Asp-Arg-ACC<br>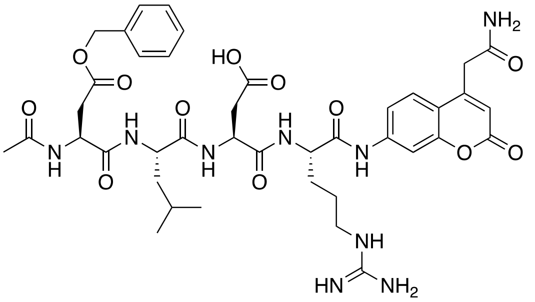

**(2)-NH-6** Ac-Nle(Obzl)-Ile-Tyr-Arg-ACC 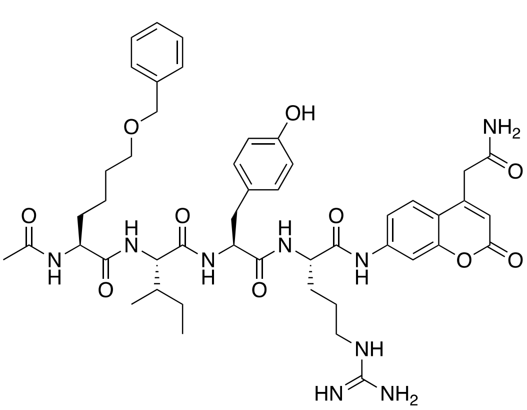	**(2)-NH-7** Ac-DTyr-Ala-hPhe-Arg-ACC 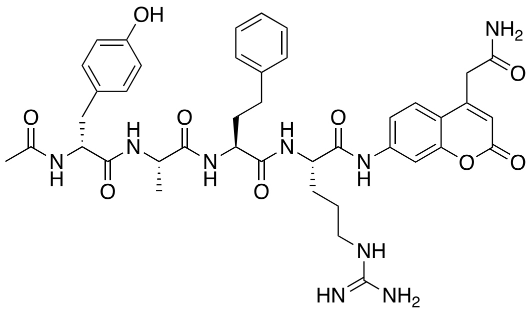
**(2)-NH-8** Ac-Asn-Val-hCha-Arg-ACC 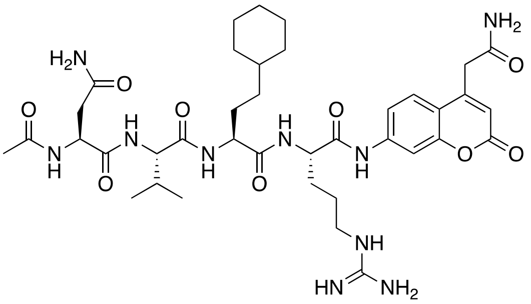	**(2)-NH-9** Ac-His-Glu(Bzl)-Leu-Arg-ACC 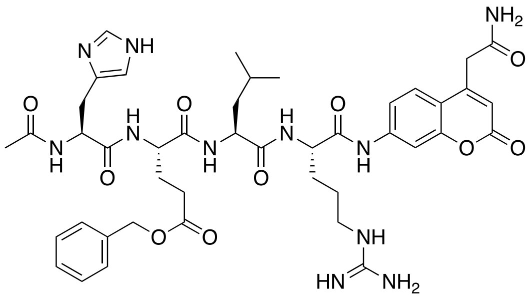
**(2)-NH-10** Ac-His-Ala-Tyr-Arg-ACC 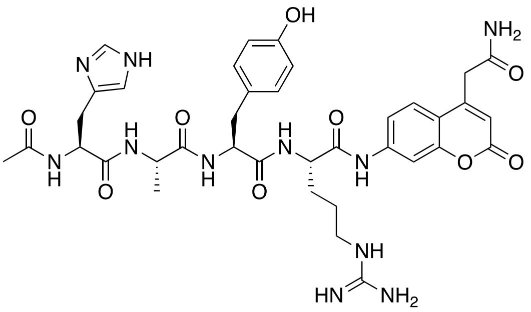	**(2)-NH-15** Ac-DTyr-Gln-hTyr-Arg-ACC 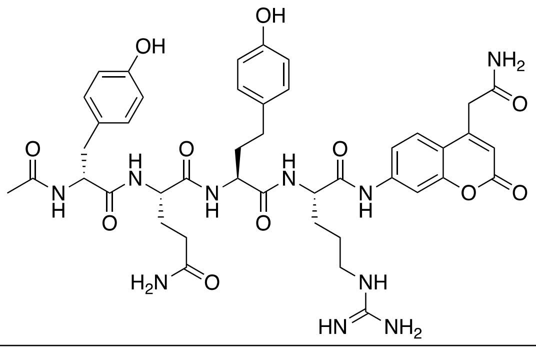
**(2)-NH-16** Ac-Hyp(Bzl)-Tyr-Phg-Arg-ACC 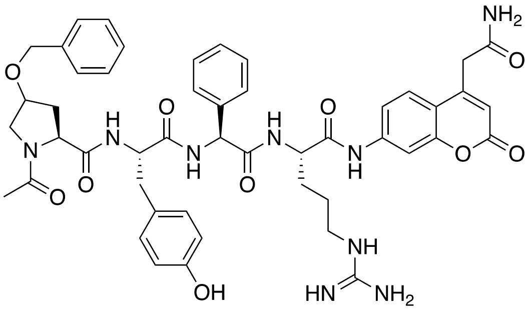	**(2)-NH-17** Ac-Nle(OBzl)-DGln-hPhe-Arg-ACC 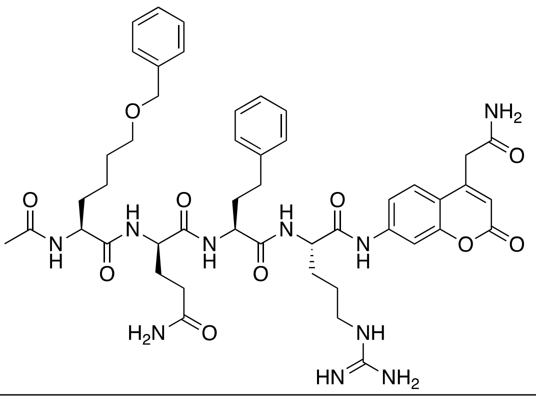
**(2)-NH-18** Ac-Asn-Bip-hCha-Arg-ACC	

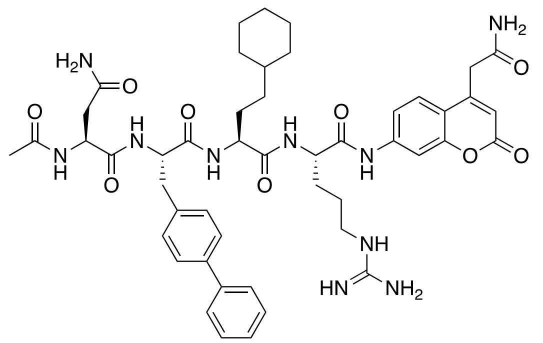

Table S2 Mass spectrometry analysis of PAD2 and PAD4 substrates. Each substrate was subjected to LC-MS analysis, and purity was confirmed by UV detection at 220 nm. All substrates showed a purity above 95%.

Code	Substrate	$[M+H]^+$ calculated	$[M+H]^+$ measured
**(4)-NH-1**	Ac-His(Bzl)-Leu-Asp-Arg-ACC	872,41	436,89 $[M+2H]^{2+}$
**(4)-NH-2**	Ac-Asp(Bzl)-DTyr-Asp-Arg-ACC	900,35	900,36
**(4)-NH-4**	Ac-hCha-Arg-Asp-Arg-ACC	855,45	428,40 $[M+2H]^{2+}$
**(4)-NH-5**	Ac-hCha-Leu-Asp-Arg-ACC	812,43	812,50
**(4)-NH-11**	Ac-Asp(Bzl)-hCha-Asp-Arg-ACC	904,42	904,50
**(4)-NH-12**	Ac-Phe(3I)-3Pal-Asp-Arg-ACC	953,24	477,33 $[M+2H]^{2+}$
**(4)-NH-13**	Ac-Phe(3I)-hCha-Asp-Arg-ACC	972,31	972,37
**(4)-NH-14**	Ac-Asp(Bzl)-Leu-Asp-Arg-ACC	850,37	850,40
**(2)-NH-6**	Ac-Nle(Obzl)-Ile-Tyr-Arg-ACC	912,46	912,55
**(2)-NH-7**	Ac-DTyr-Ala-hPhe-Arg-ACC	812,37	812,38
**(2)-NH-8**	Ac-Asn-Val-hCha-Arg-ACC	797,43	798,48
**(2)-NH-9**	Ac-His-Glu(Bzl)-Leu-Arg-ACC	886,42	443,87 $[M+2H]^{2+}$
**(2)-NH-10**	Ac-His-Ala-Tyr-Arg-ACC	788,35	394,72 $[M+2H]^{2+}$
**(2)-NH-15**	Ac-DTyr-Gln-hTyr-Arg-ACC	885,39	885,44
**(2)-NH-16**	Ac-Hyp(Bzl)-Tyr-Phg-Arg-ACC	916,40	916,47
**(2)-NH-17**	Ac-Nle(OBzl)-DGln-hPhe-Arg-ACC	925,46	925,55
**(2)-NH-18**	Ac-Asn-Bip-hCha-Arg-ACC	921,46	921,55

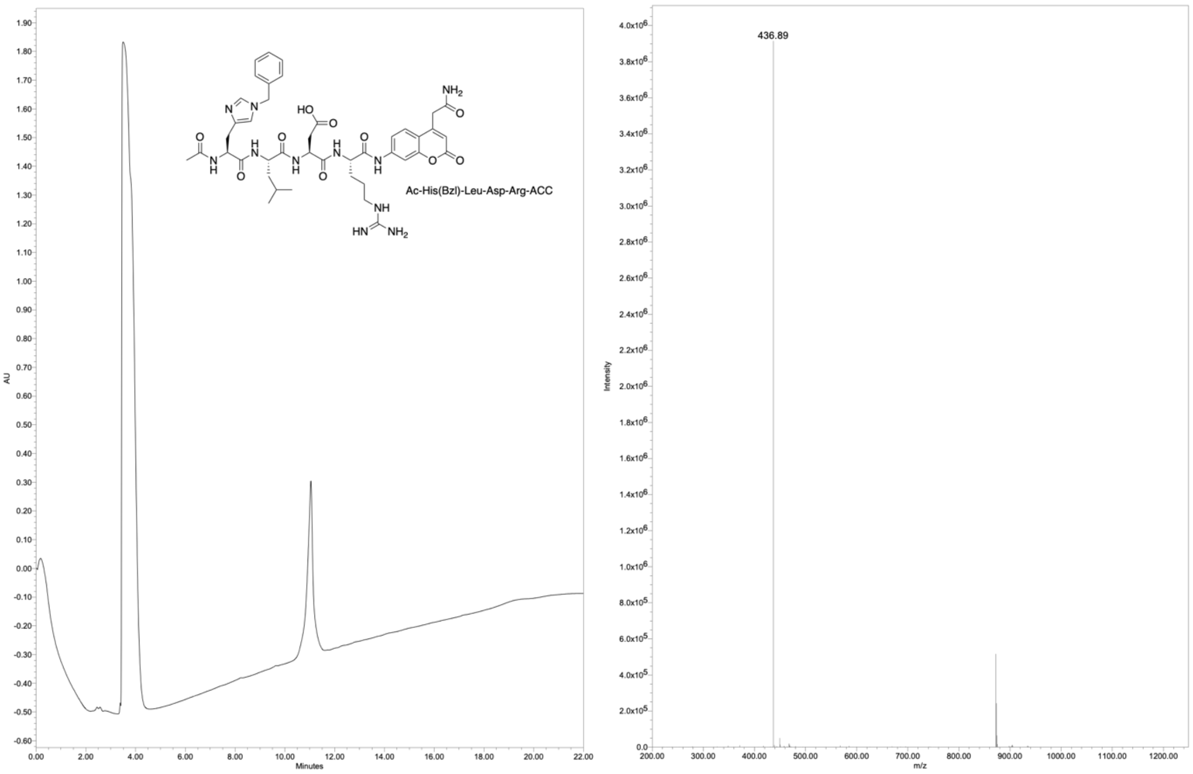

Figure S3 LC-MS analysis of the PAD4 substrate Ac-His(Bzl)-Leu-Asp-Arg-ACC, (4)-NH-1. Purity was assessed by liquid chromatography (UV detection at 220nm; left), and identity was confirmed by mass spectrometry (m/z; right).

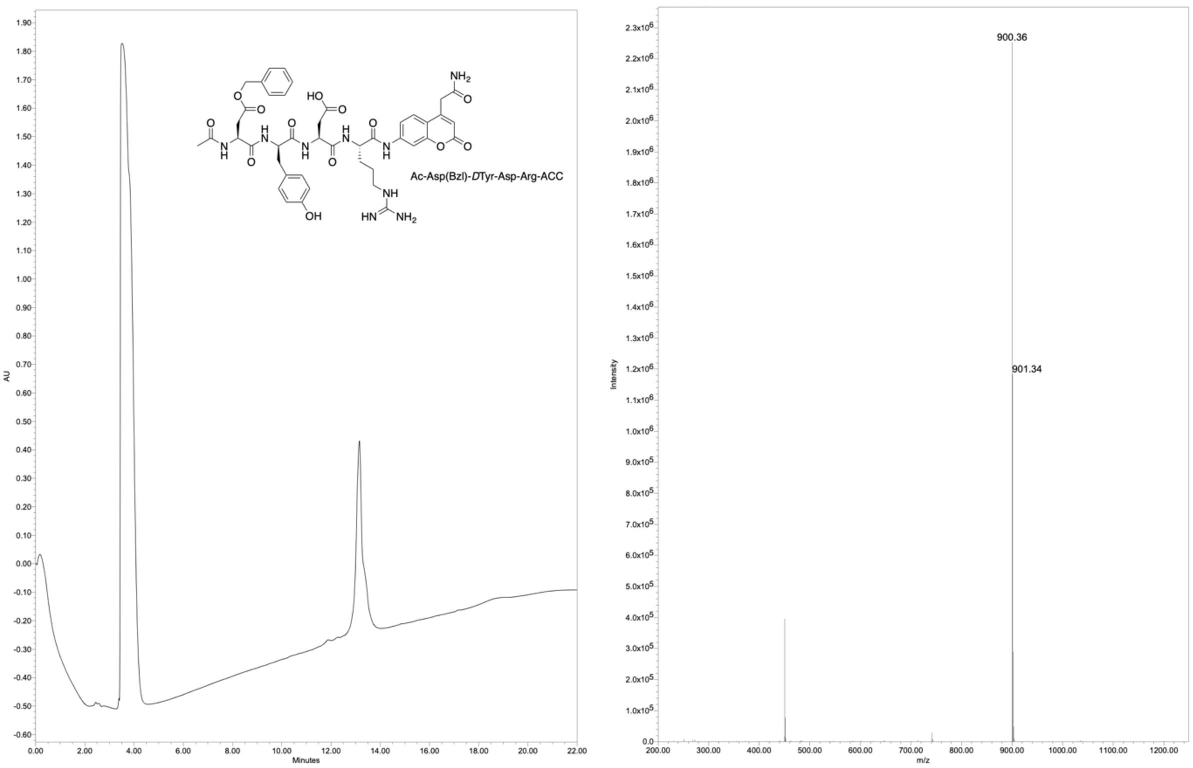

Figure S4 LC-MS analysis of the PAD4 substrate Ac-Asp(Bzl)-DTyr-Asp-Arg-ACC, (4)-NH-2. Purity was assessed by liquid chromatography (UV detection at 220nm; left), and identity was confirmed by mass spectrometry (m/z; right).

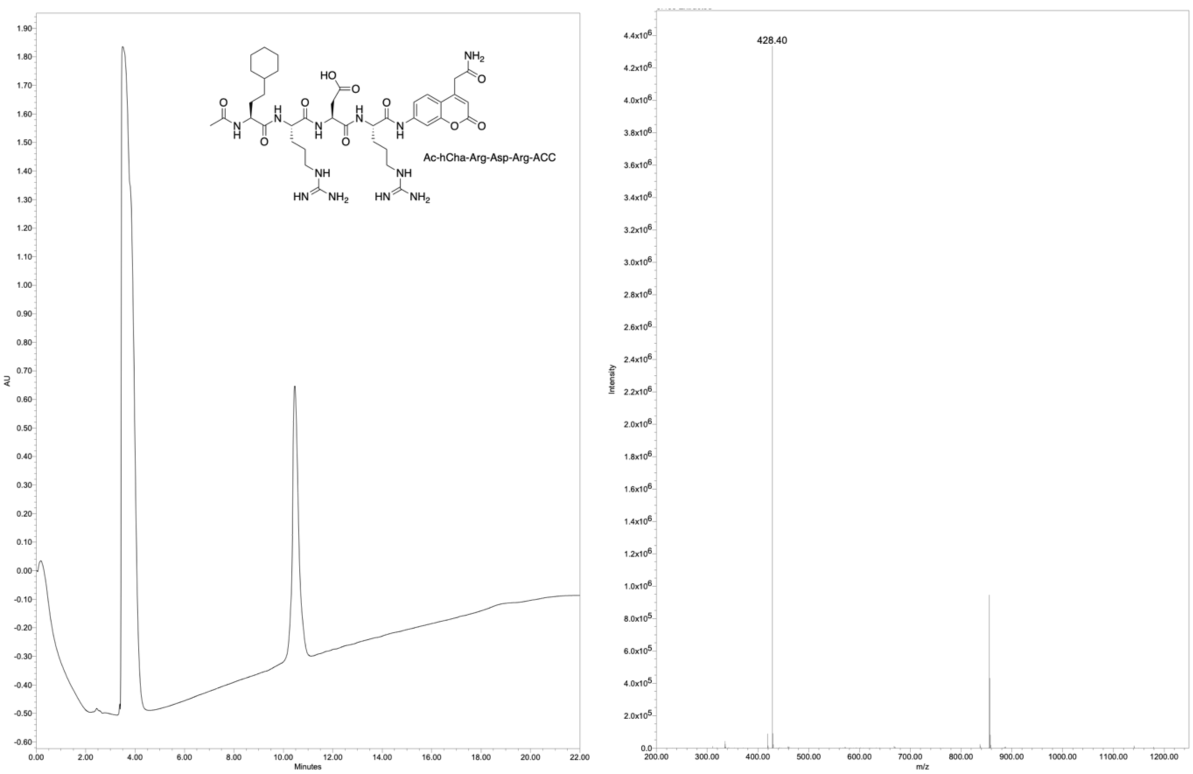

Figure S5 LC-MS analysis of the PAD4 substrate Ac-hCha-Arg-Asp-Arg-ACC, (4)-NH-4. Purity was assessed by liquid chromatography (UV detection at 220nm; left), and identity was confirmed by mass spectrometry (m/z; right).

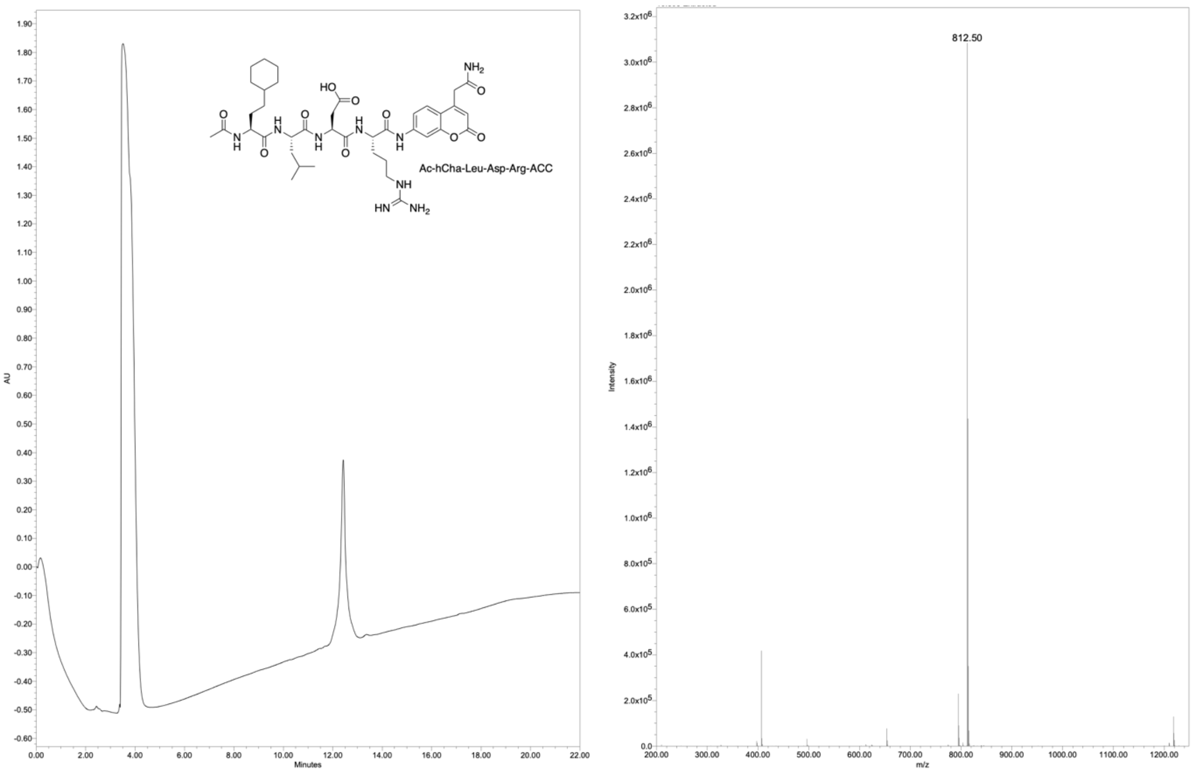

Figure S6 LC-MS analysis of the PAD4 substrate Ac-hCha-Leu-Asp-Arg-ACC, (4)-NH-5. Purity was assessed by liquid chromatography (UV detection at 220nm; left), and identity was confirmed by mass spectrometry (m/z; right).

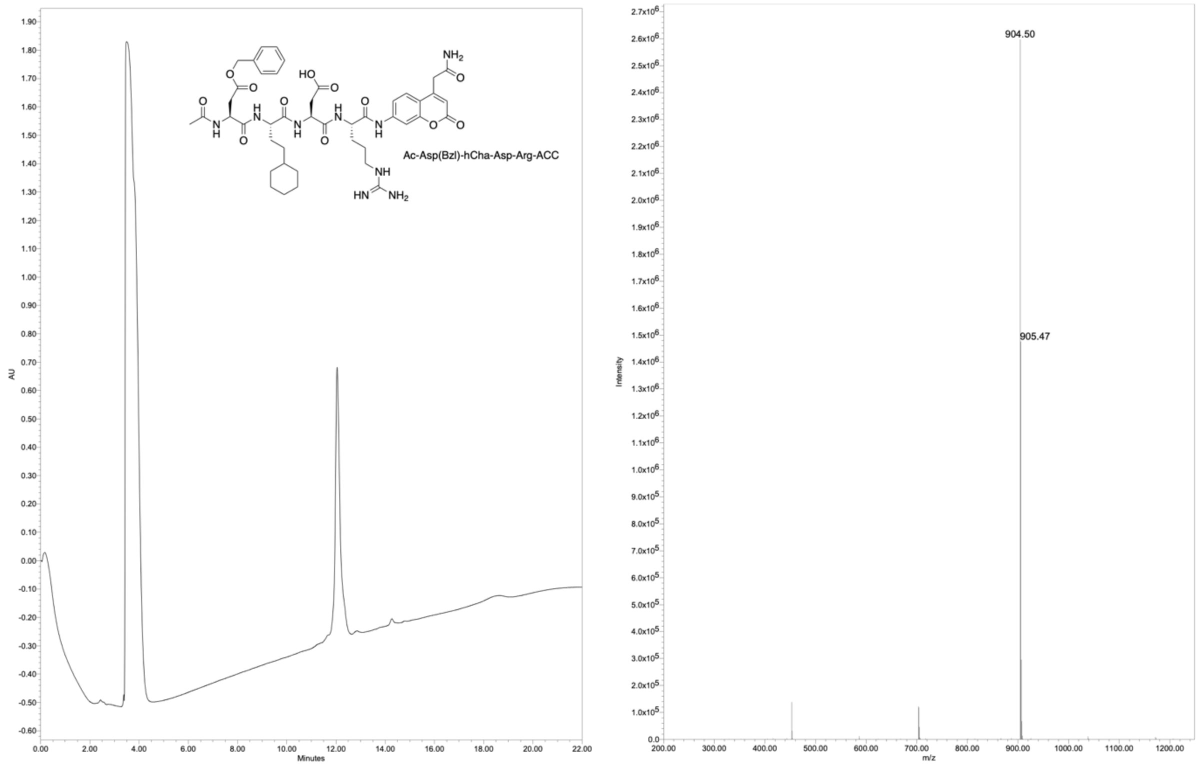

Figure S7 LC-MS analysis of the PAD4 substrate Ac-Asp(Bzl)-hCha-Asp-Arg-ACC, (4)-NH-11. Purity was assessed by liquid chromatography (UV detection at 220nm; left), and identity was confirmed by mass spectrometry (m/z; right).

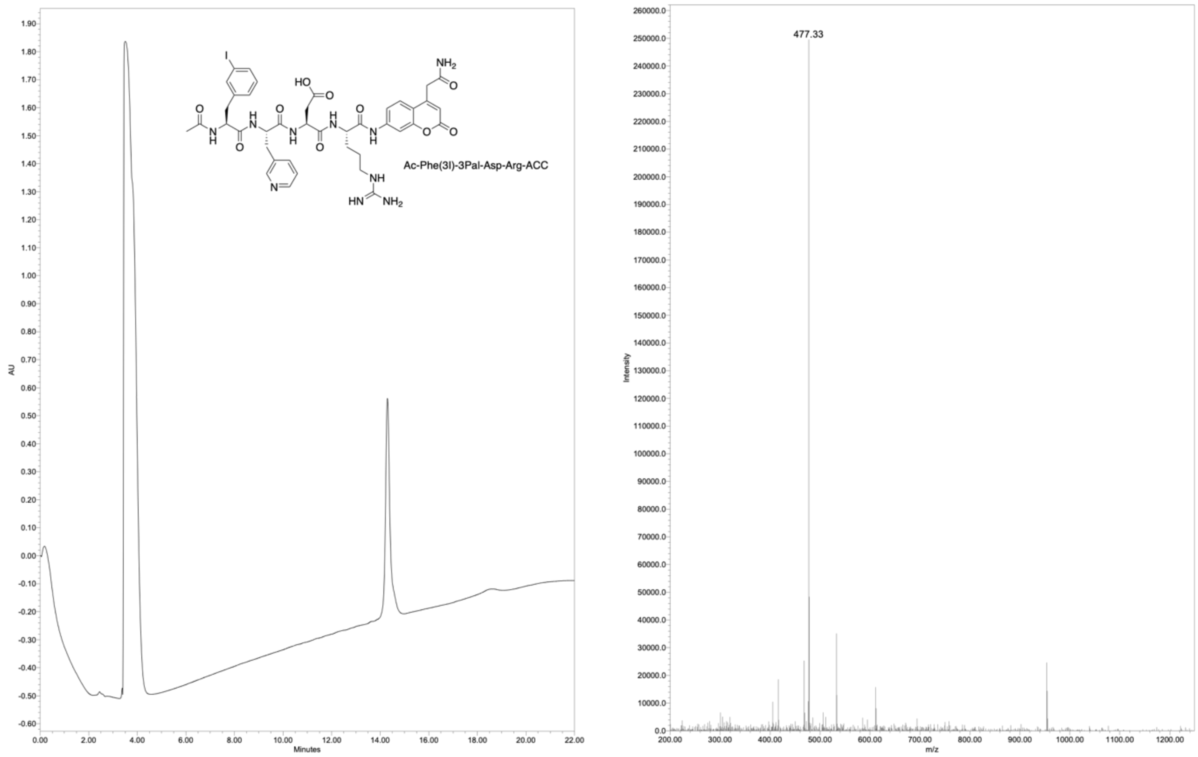

Figure S8 LC-MS analysis of the PAD4 substrate Ac-Phe(3I)-3Pal-Asp-Arg-ACC, (4)-NH-12. Purity was assessed by liquid chromatography (UV detection at 220nm; left), and identity was confirmed by mass spectrometry (m/z; right).

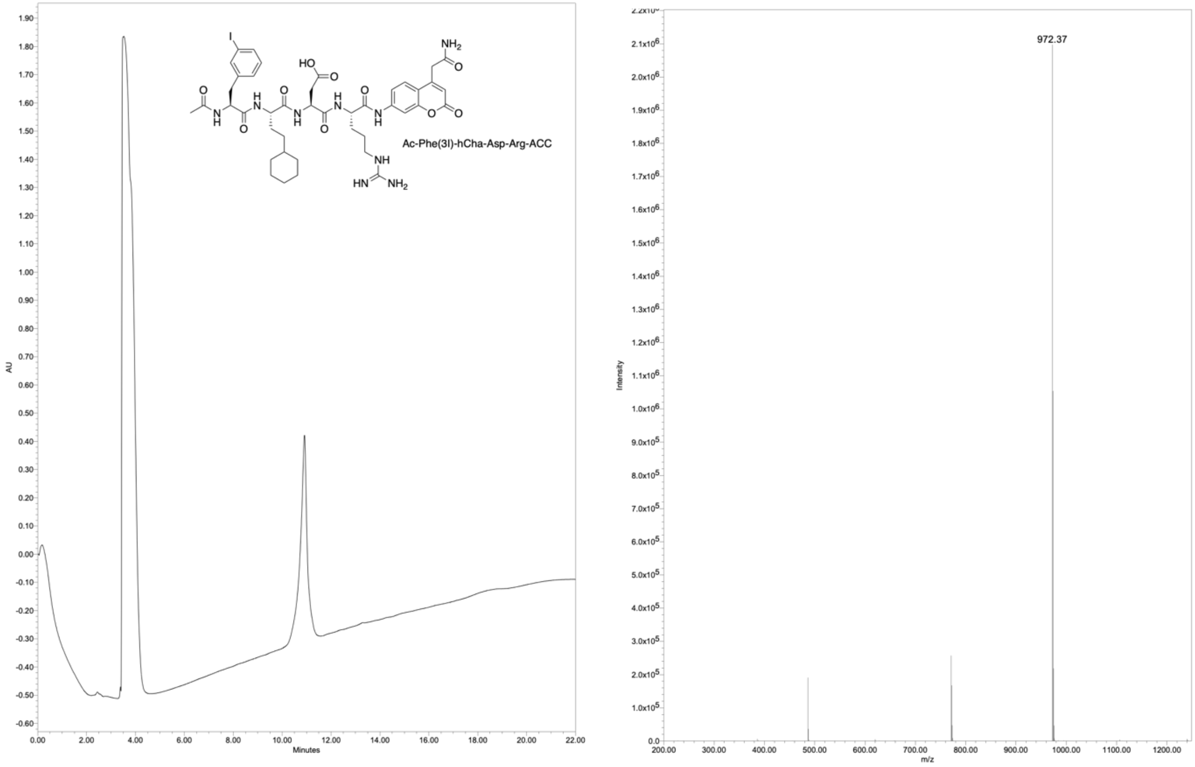

Figure S9 LC-MS analysis of the PAD4 substrate Ac-Phe(3I)-hCha-Asp-Arg-ACC, (4)-NH-13. Purity was assessed by liquid chromatography (UV detection at 220nm; left), and identity was confirmed by mass spectrometry (m/z; right).

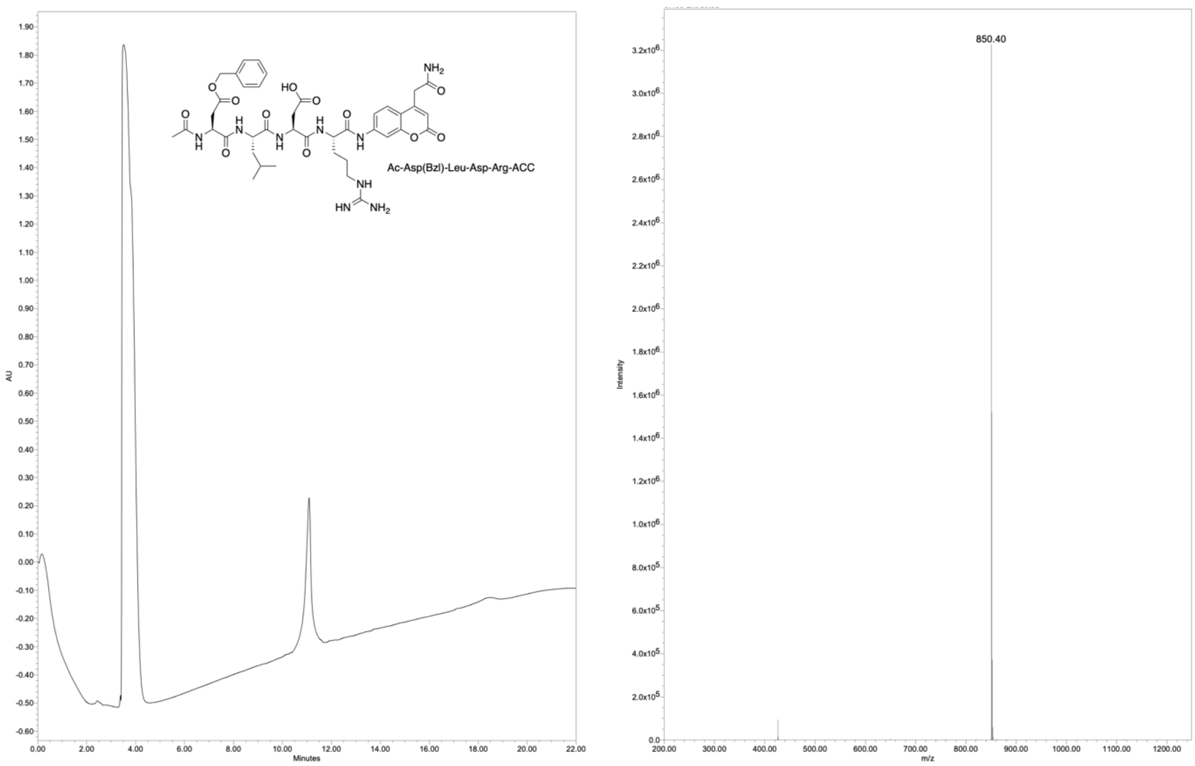

Figure S10 LC-MS analysis of the PAD4 substrate Ac-Asp(Bzl)-Leu-Asp-Arg-ACC, (4)-NH-14. Purity was assessed by liquid chromatography (UV detection at 220nm; left), and identity was confirmed by mass spectrometry (m/z; right).

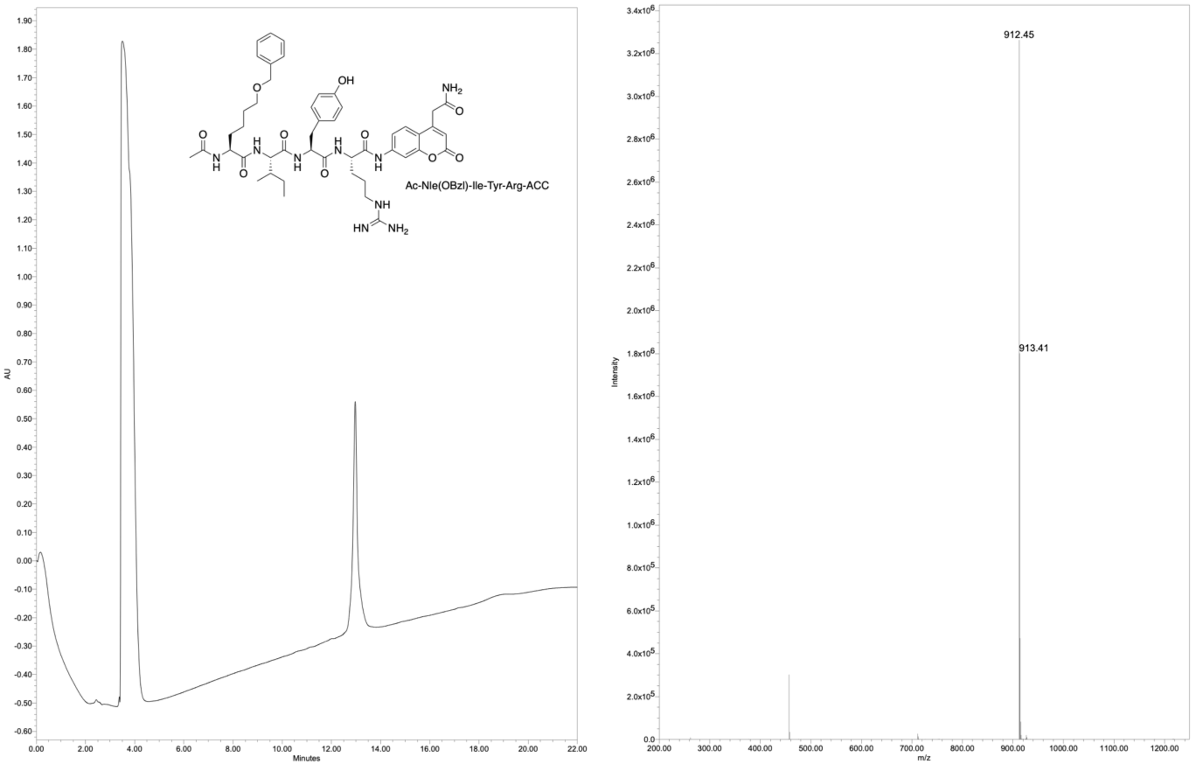

Figure S11 LC-MS analysis of the PAD2 substrate Ac-Nle(OBzl)-Ile-Tyr-Arg-ACC, (2)-NH-6. Purity was assessed by liquid chromatography (UV detection at 220nm; left), and identity was confirmed by mass spectrometry (m/z; right).

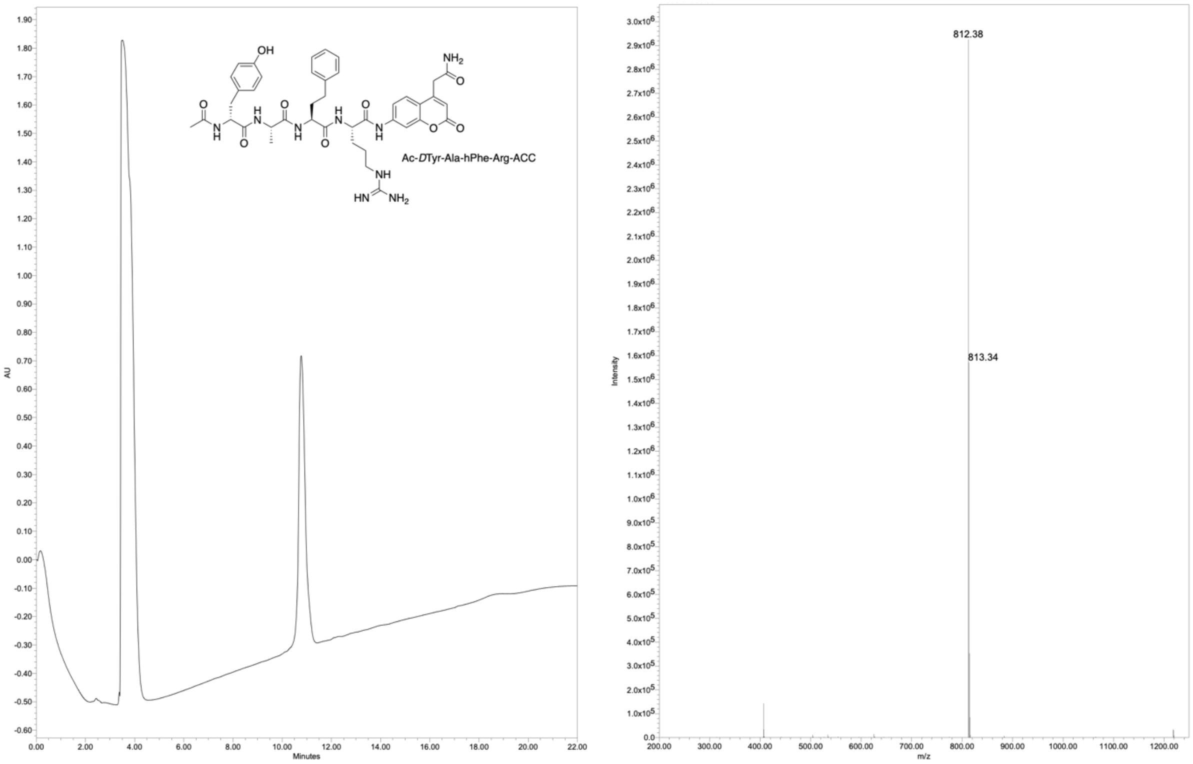

Figure S12 LC-MS analysis of the PAD2 substrate Ac-DTyr-Ala-hPhe-Arg-ACC, (2)-NH-7. Purity was assessed by liquid chromatography (UV detection at 220nm; left), and identity was confirmed by mass spectrometry (m/z; right).

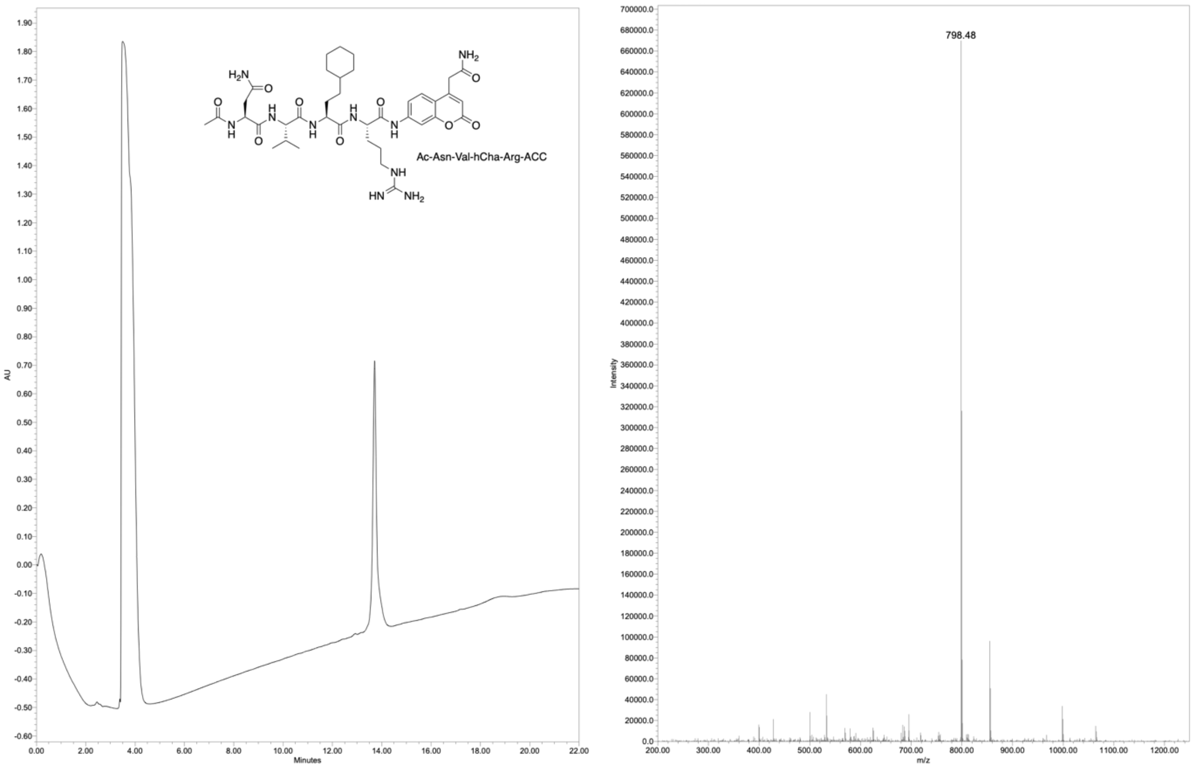

Figure S13 LC-MS analysis of the PAD2 substrate Ac-Asn-Val-hCha-Arg-ACC, (2)-NH-8. Purity was assessed by liquid chromatography (UV detection at 220nm; left), and identity was confirmed by mass spectrometry (m/z; right).

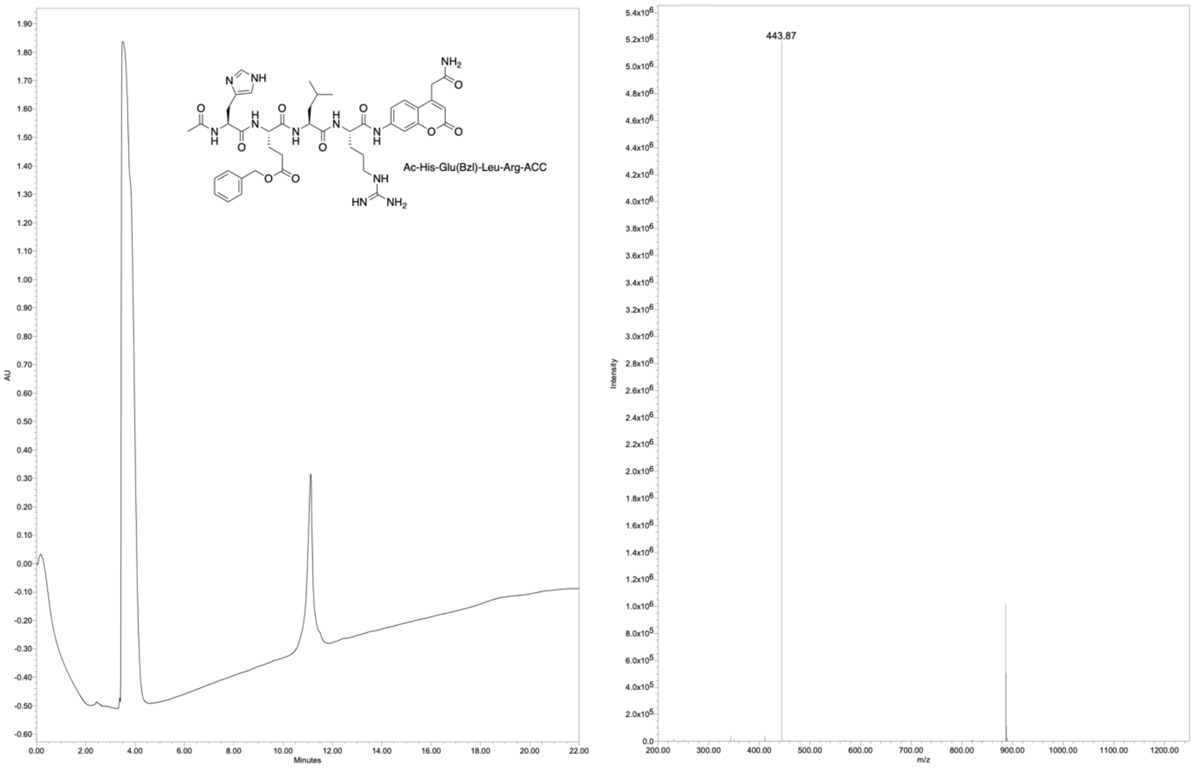

Figure S14 LC-MS analysis of the PAD2 substrate Ac-His-Glu(Bzl)-Leu-Arg-ACC, (2)-NH-9. Purity was assessed by liquid chromatography (UV detection at 220nm; left), and identity was confirmed by mass spectrometry (m/z; right).

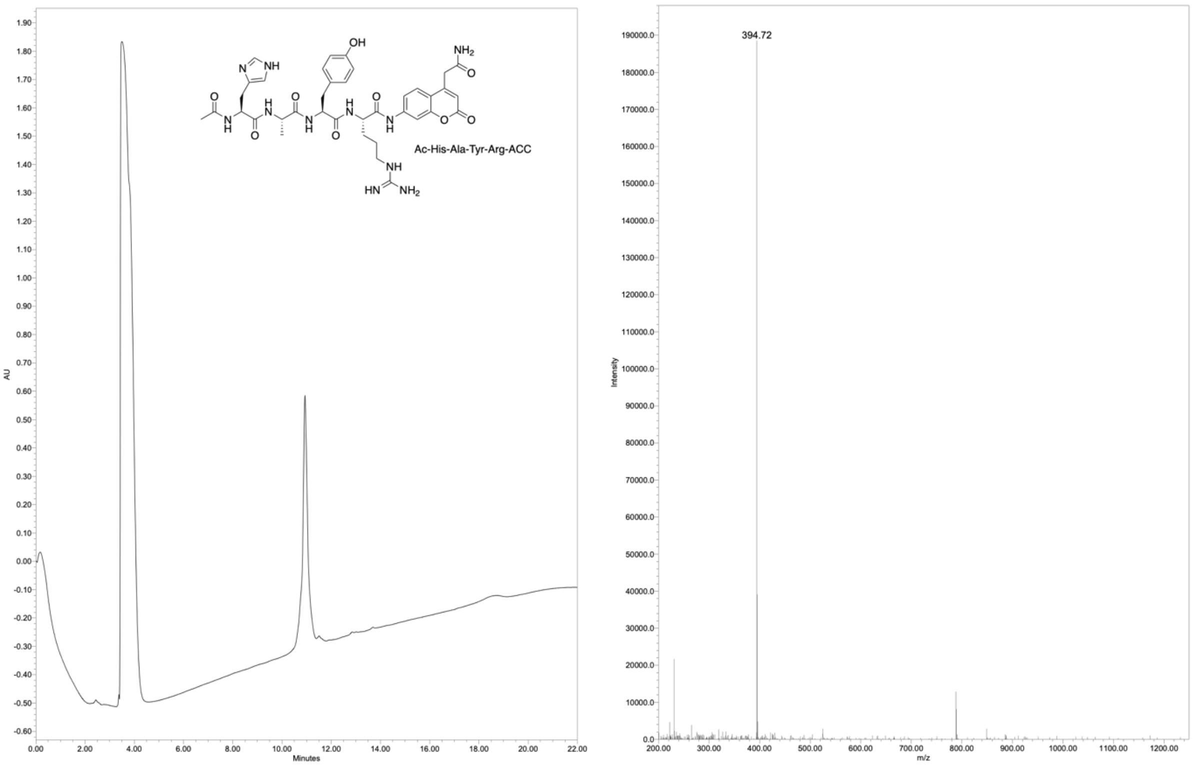

Figure S15 LC-MS analysis of the PAD2 substrate Ac-His-Ala-Tyr-Arg-ACC, (2)-NH-10. Purity was assessed by liquid chromatography (UV detection at 220nm; left), and identity was confirmed by mass spectrometry (m/z; right).

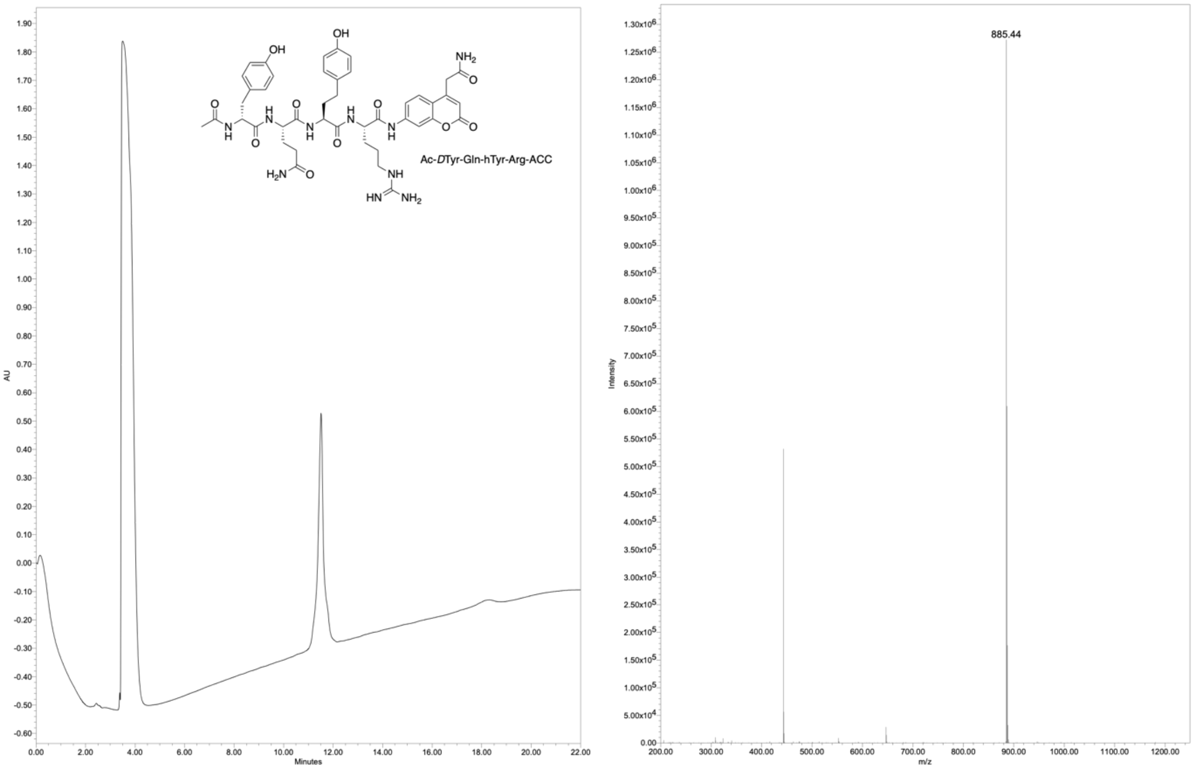

Figure S16 LC-MS analysis of the PAD2 substrate Ac-DTyr-Gln-hTyr-Arg-ACC, (2)-NH-15. Purity was assessed by liquid chromatography (UV detection at 220nm; left), and identity was confirmed by mass spectrometry (m/z; right).

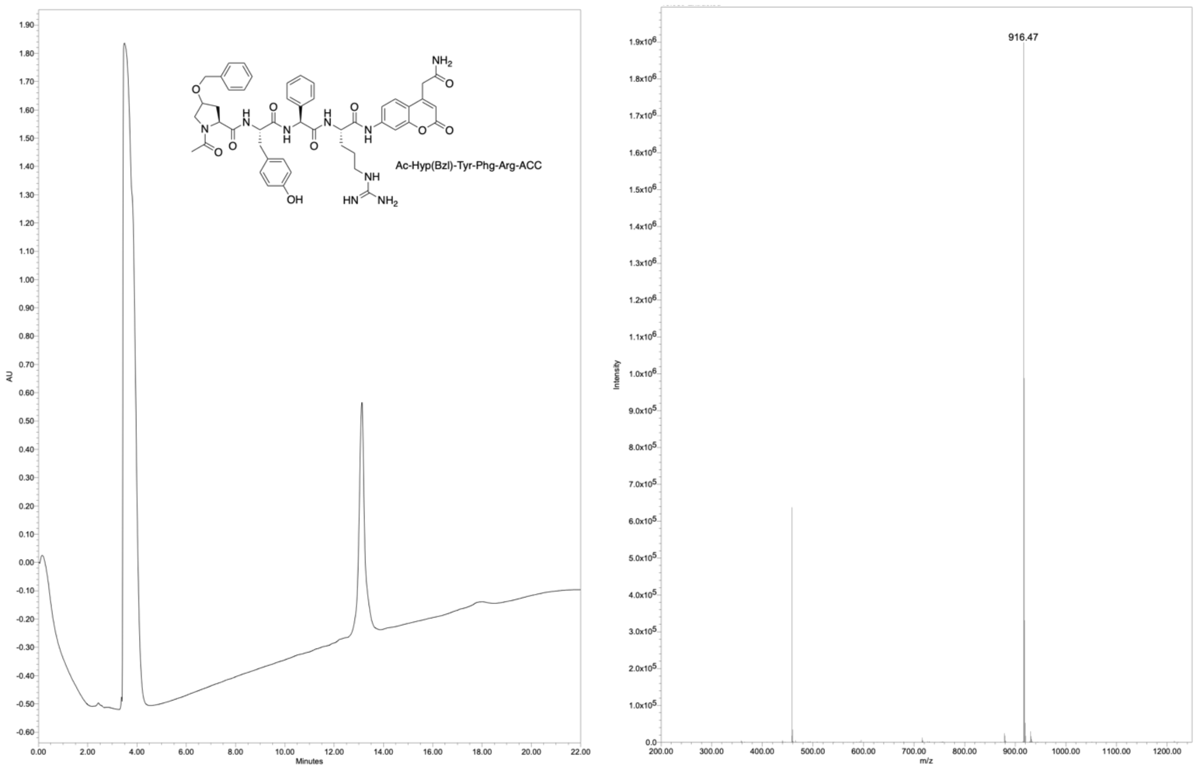

Figure S17 LC-MS analysis of the PAD2 substrate Ac-Hyp(Bzl)-Tyr-Phg-Arg-ACC, (2)-NH-16. Purity was assessed by liquid chromatography (UV detection at 220nm; left), and identity was confirmed by mass spectrometry (m/z; right).

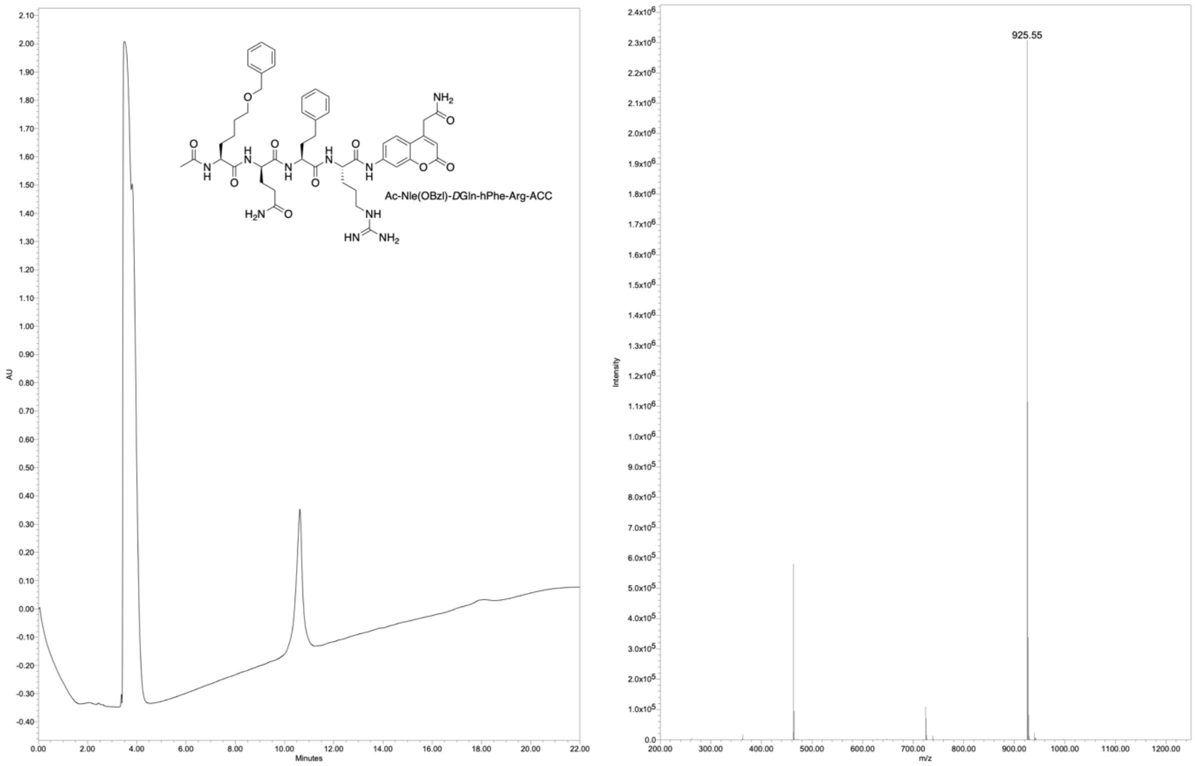

Figure S18 LC-MS analysis of the PAD2 substrate Ac-Nle(OBzl)-DGln-hPhe-Arg-ACC, (2)-NH-17. Purity was assessed by liquid chromatography (UV detection at 220nm; left), and identity was confirmed by mass spectrometry (m/z; right).

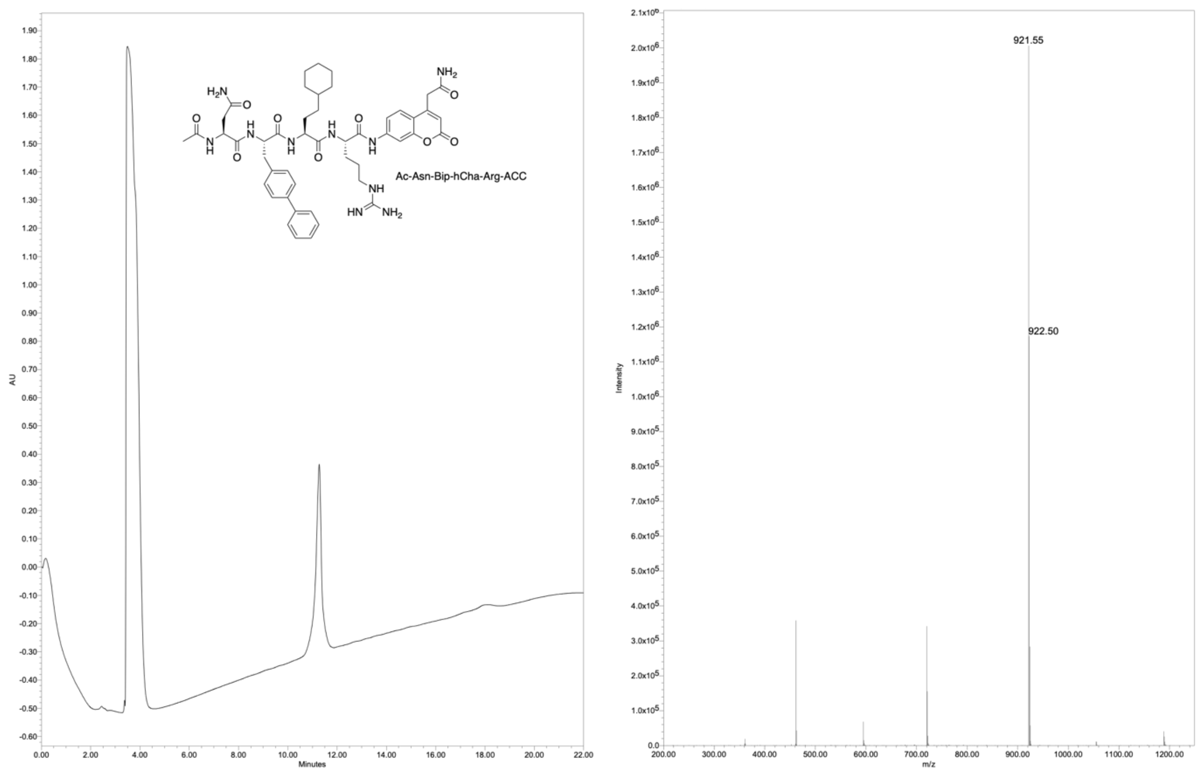

Figure S19 LC-MS analysis of the PAD2 substrate Ac-Asn-Bip-hCha-Arg-ACC, (2)-NH-18. Purity was assessed by liquid chromatography (UV detection at 220nm; left), and identity was confirmed by mass spectrometry (m/z; right).
